# Dissecting the genetics of forage quality traits in soft red winter wheat in the U.S. southeast region

**DOI:** 10.3389/fpls.2026.1893113

**Published:** 2026-08-26

**Authors:** Maria Ali, Ali Missaoui, Gina Brown-Guedira, Uttam Saha, Mohamed Mergoum

**Affiliations:** 1 Department of Crop and Soil Sciences, University of Georgia, Griffin, GA, United States; 2 USDA-ARS Plant Science Research, Raleigh, NC, United States; 3 Agricultural and Environmental Services Laboratories, University of Georgia Cooperative Extension, Athens, GA, United States

**Keywords:** dual-purpose wheat, forage quality, GWAS, marker-assisted selection, QTL

## Abstract

Winter wheat plays a viable role in agriculture, not only as a primary grain crop but also as a valuable forage source that bridges fall-spring forage gaps in many regions, including the southeastern (SE) U.S. Despite its nutritive potential, genetic basis of forage-quality traits remains insufficiently understood, limiting breeding efforts for dual-purpose cultivars. This study aimed to dissect the genetic architecture of forage quality in 182 soft red winter wheat (SRWW) genotypes adapted to the SE U.S. using genome-wide association study (GWAS). Field experiments were carried out in randomized complete block design across three Georgia locations over two growing seasons (2023-2025), with forage sampled at the end of tillering and evaluated using near-infrared reflectance spectroscopy. Significant phenotypic variation was observed for dry matter (DM), crude protein (CP), acid and neutral detergent fiber (ADF, NDF), acid detergent lignin (ADL), total digestible nutrients (TDN), sugars (SUG), and relative forage quality (RFQ). Heritability estimates ranged from low-to-moderate in combined environments and from low-to-high within individual locations. Correlation analysis revealed strong positive associations among fiber-related traits and negative associations with TDN, RFQ, and SUG, while CP declined with increasing fiber. Genome-wide association analysis identified 282 significant marker-trait associations (P < 1×10^-4^) across 19 chromosomes, which were consolidated into 121 QTLs, including 27 major-effect QTLs. Three QTLs *QRfq.uga-3B.1*, *QRfq.uga-3B.2* (RFQ) and *QDm/Sug.uga-7A* (DM, SUG) were stable across locations while *QAdf/Adl.uga-2A* (ADF, ADL) and *QDm/Sug.uga-7A* (DM, SUG) indicated multi-trait control. Notably, 25 of the 27 major QTLs were putatively novel, highlighting substantial untapped allelic diversity for forage-quality improvement in SE SRWW. Favorable allele accumulation resulted in an overall improvement in forage quality, increasing desirable nutritive traits (DM, RFQ, SUG, CP) while reducing undesirable traits (ADF, ADL). Candidate gene analysis linked six major QTLs with genes implicated in abiotic stress response, plant development, and metabolic regulation, supporting their functional relevance in forage-quality determination. Incorporating these loci into breeding programs provides a robust genetic framework for marker-assisted selection, enabling the development of dual-purpose wheat cultivars with enhanced forage quality, thereby strengthening wheat’s utility as a reliable forage resource during periods of seasonal feed scarcity in SE production systems.

## 1 Introduction

Wheat (*Triticum aestivum* L.) serves as a global staple food, primarily cultivated as a grain crop, but in mixed crop-livestock systems it can also serve as an important dual-purpose cereal by supplying forage before grain harvest (Severino da Silva et al., 2026). This role is especially relevant in the southeastern (SE) U.S., where livestock systems rely heavily on warm-season perennial grasses such as bermudagrass and bahiagrass leave a predictable forage gap from approximately November to April (Baxter and Singleton, 2025; Severino da Silva et al., 2026). Annual cool-season grasses can bridge this gap and lessen dependence on stored forage or supplementation when perennial pastures are least productive (Baxter and Singleton, 2025). Feed supply conditions also remain tight at the national level, as U.S. hay stocks in May 2026, were 23.3 million tons, which was 3.3% below the previous year (USDA National Agricultural Statistics Service, 2026). The regional livestock base further underscores the practical relevance of winter forage resources, with substantial cattle inventories across Alabama, Georgia, North Carolina, and South Carolina. Wheat also remains an economically relevant crop in the SE region, where current acreage still contributes meaningful farm-gate value and where dual-purpose management offers an opportunity to increase returns from existing wheat production systems (USDA National Agricultural Statistics Service, 2025a, 2025b, 2025c, 2025d).

Recent research also indicates that dual-purpose wheat can be viable in the SE, where wheat has historically been used less frequently as dual-purpose than in the Southern Great Plains (Hinson et al., 2023; West et al., 2024; Severino da Silva et al., 2026). West et al. (2024) reported cultivar-dependent differences in forage biomass, nutritive value, and grain yield under dual-purpose management, demonstrating that genotype strongly influences system performance. In a more recent southeastern study, Severino da Silva et al. (2026) found that selected small-grain cultivars produced 4.14 to 5.46 Mg ha^-^¹ seasonal forage accumulation and concluded that dual-purpose management can increase producer flexibility, despite grain-yield penalties relative to grain-only production. These considerations are especially important in soft red winter wheat (SRWW), the dominant market class in much of the southeastern U.S., where added forage value could strengthen the role of wheat in diversified production systems (USDA Economic Research Service, 2026).

Despite this agronomic and economic importance, the genetics of wheat forage quality remain much less developed than the genetics literature devoted to grain yield, disease resistance, and end-use quality (Siahsar et al., 2009; Saini et al., 2022; Subedi et al., 2023). Earlier wheat studies provide proof of concept, but they have mostly focused on straw and post-harvest biomass traits. Bellucci et al. (2015) used association mapping and identified six QTL linked to grain yield, plant height, and sugars released after straw enzymatic hydrolysis. Joshi et al. (2019) identified genomic regions for straw fodder-quality traits such as ADF, ADL, NDF, IVOMD, and metabolizable energy in a panel of 287 diverse spring wheat lines. Esposito et al. (2022) subsequently applied multi-locus genome-wide association study (GWAS) in tetraploid wheat and identified loci for ADF, ADL, and NDF-related variation. Jensen et al. (2011) observed genetic variation in traits such as wheat straw enzymatic digestibility and Lindedam et al. (2012) reported sugar release after pre-treatment and enzymatic hydrolysis, indicating potential for further exploration of wheat forage quality-related traits through GWAS. Similar progress has been reported in related cereals: De Zutter et al. (2024) identified digestibility-associated MTAs and candidate genes in triticale forage, Nguyen et al. (2020) detected stable QTLs and candidate genes for digestibility and lignin content in rice straw, and Lu et al. (2025) used combined GWAS and meta-analysis to prioritize quality-related genomic regions and candidate genes in silage maize.

Taken together, these studies reveal a clear knowledge gap for dual-purpose wheat breeding in the SE U.S. Most of the prior work has targeted straw digestibility, saccharification, silage quality, or biomass composition rather than fresh forage harvested at a livestock-relevant vegetative stage (Bellucci et al., 2015; Joshi et al., 2019; Esposito et al., 2022; Nguyen et al., 2020; De Zutter et al., 2024; Lu et al., 2025). In addition, previous association mapping panels have predominantly focused on spring wheat, tetraploid wheat, Scandinavian winter wheat, or other cereal populations rather than SRWW adapted to the SE U.S. As a result, little is known about the loci controlling fresh forage quality in this target germplasm, the stability of those loci across southeastern environments, or the candidate genes most useful for downstream breeding and validation.

GWAS is particularly well suited to this problem because forage-quality traits are complex, quantitatively inherited, and strongly influenced by environment. GWAS exploits historical recombination across diverse population, enabling the detection of marker-trait associations (MTAs) at higher mapping resolution and capturing a broader allelic spectrum than biparental QTL mapping, thereby often provides finer mapping resolution (Alqudah et al., 2020). Post-GWAS analyses further strengthen its value by prioritizing candidate genes, reduce false positive association through integration with complementary genomic data, and linking marker discovery to functional validation and marker-assisted breeding (Nguyen et al., 2020; Kaňovská et al., 2024).

The novelty of the present study lies in combining multi-environment phenotyping of fresh forage quality with GWAS in a SE U.S. adapted SRWW diversity panel. The SE region provides suitable environmental conditions conducive to cereal grain production, with mild winters and adequate rainfall (Sulc and Franzluebbers, 2014). Dissecting wheat forage quality and highlighting its importance during fall forage scarcity could provide SE producers with practical and beneficial economic alternatives. This study aimed to dissect the genetic architecture of forage-quality traits in SRWW and to identify loci and candidate genes that can support the development of improved dual-purpose cultivars. Specifically, the objectives were to: (i) quantify phenotypic variation and trait relationships among key forage-quality traits across environments; (ii) identify significant MTAs, major QTL, including novel and stable loci controlling these traits; and (iii) determine the candidate genes underlying major loci to facilitate marker-assisted selection and future functional validation.

## 2 Materials and methods

### 2.1 Plant material and experimental layout

A GWAS panel of 182 genotypes was used to evaluate the forage-quality traits in wheat. It includes elite lines of SRWW developed from several SE breeding programs, including Arkansas (AR), Florida (FL), Georgia (GA), Louisiana (LA), North Carolina (NC), South Carolina (SC), Texas (TX), and Virginia (VA) using a modified pedigree method. This includes single plant selection in F2, F4, and F5 while F3 was advanced in a winter nursery using a bulk method. The panel was grown alongside four high yielding check varieties: AGS3030, AGS2033, AGS4043, and Hilliard. AGS3030, AGS2033, and AGS4043 were developed and released by the University of Georgia (UGA) Small Grains Breeding and Genetics Program, suitable for both grain and forage production statewide in GA (Mailhot et al., 2024). Hilliard is a late-maturing cultivar released by Virginia Tech with good pest and disease resistance and good grain-yield performance in GA.

The field experiments were laid out in a randomized complete block design (RCBD) with two replications at three GA locations over two growing seasons (2023-2025): the UGA Southwestern Research and Education Center (SWREC) in Plains, GA (32° 2.80’ N, 84° 21.98’ W), during 2023–2024 and 2024-2025; Iron Horse Farm (IHF) in Watkinsville, GA (33° 51.5’ N, 83° 23.5’ W), and J. Phil Campbell Sr. (JPC) Research and Education Center in Athens, GA (33° 52.2’ N, 83° 25.8’ W) during 2024-2025. SWREC location is characterized by extremely low temperatures of 10-15°F, annual precipitation of 46–50 inches, and predominantly Greenville sandy clay loam soils. The JPC site is located in the GA Piedmont and characterized by low temperatures of 10-15°F and an average annual rainfall of 48 inches. The soil across JPC varies significantly, encompassing Cecil sandy loam, Wickham sandy loam, and Chewacla fine loam. Similarly, IHF is characterized by Pacolet sandy clay loam soil with a moderate slope typical of the regional Piedmont and receives approximately 123 cm annual rainfall (Mahmud et al., 2021). These site-year combinations were referred to as GAP-24/25, IHF-25, and JPC-25, respectively, in this study. The locations were selected to represent contrasting but relevant agro-ecological conditions within Georgia so that environmental main effects and genotype × environment interaction (G x E) could be estimated within a target population of southeastern production environments. Each entry was grown in a plot measuring 7 rows by 3m long, with 7 inches between rows.

Planting typically occurred during November at a seed rate of 65 g plot^-1^. A GPS-equipped tractor (John Deere, Moline, Illinois, USA), and Trimble Technologies (Westminster, CO, USA), and an auto trip seed drill (Hege Equipment Inc, Salt Lake City, UT, USA) were used for planting. Pre-plant fertilization included 78 lb Nitrogen (N) and 12 lb Sulfur (S) per acre. Post-planting fertilizer applications were tailored to the requirements of the dual-purpose system, with 50 lb N and 10 lb S per acre applied after the forage biomass harvest to support grain recovery following vegetative forage removal. Thus, the panel was evaluated under dual-purpose management rather than forage-only management. The agronomic management was carried out under standard practices overseen by the field management team. To minimize border effects and experimental error, forage samples were collected from the internal harvest area of each plot and border rows/plot ends were excluded from biomass sampling. The use of two replications reflects a practical resource-allocation trade-off in multi-environment trials: once within-site precision is adequate, allocating finite resources to a broader and more representative sample of environments can improve the reliability of genotype evaluation and strengthen inference on genotype performance across the target population of environments (Talbot, 1997).

### 2.2 Phenotypic data collection

Forage samples for quality analysis were collected by hand clipping approximately 10 cm above the soil surface at the end of tillering and before the stem elongation stage i.e. Zadoks growth stage 31 (Zadoks et al., 1974) or the development of the first hollow stem (Edwards and Horn, 2010). Zadoks growth stage 31 was selected because it represents a key transition in dual-purpose wheat, where forage remains vegetative and highly nutritive, and harvest at or before first hollow stem supports subsequent grain yield potential. Sampling was conducted at the target developmental stage in each environment rather than on a fixed date to ensure consistent plant developmental stage across environments. Approximately 500 g of fresh biomass was sampled per plot from a defined internal sampling area of 1 foot long 2 rows and weighed, excluding border rows and plot ends. Samples were then dried at 60 °C in an air-circulation oven for 48 h and reweighed for DM. The DM was calculated by dividing the dry forage weight by the fresh forage weight. Dried samples were ground in a Thomas-Willy Laboratory Mill Model 4 (Arthur H Thomas Company, Philadelphia, PA) to achieve a particle size passing through a 1 mm screen, consistent with standard NIRS forage-sample preparation protocols. Ground samples were homogenized and stored in sealed plastic clip-lock bags to minimize post-drying moisture uptake before analysis.

For NIRS analysis, each sample was scanned 32 times using the FOSS DS3 NIR system (FOSS North America, Eden Prairie, Minnesota, USA) in reflectance mode, and the average spectrum was used for prediction. 16 scans of an internal standard ceramic disk were performed before and after each batch to ensure instrument stability and calibration. Approximately 5 g of sample was packed into ring cups (Part# IH-0386) and secured with a paper backing for scanning. Spectra were analyzed using the 25GH50.EQA calibration equation from the NIRS Forage and Feed Testing Consortium (https://www.nirsconsortium.com/). which has been validated for major forage-quality traits, including DM, crude protein (CP), acid detergent fiber (ADF), neutral detergent fiber (NDF), acid detergent lignin (ADL), total digestible nutrients (TDN), sugars (SUG), and relative forage quality (RFQ). Calibration and validation statistics, including RSQ and standard error of cross-validation, were verified, and replicate scans improved prediction precision. Quality-control measures included verification of sample dryness, uniform particle size, careful cup packing, instrument diagnostics, and rescanning of flagged samples to ensure accurate and reliable predictions.

### 2.3 Phenotypic data analysis

The phenotypic data analysis was conducted using R (RStudio, Boston, MA, USA). Five datasets were prepared including GAP-24/25, IHF-25, JPC-25, and All-combined (GAP-24/25, IHF-25). Analysis of variance (ANOVA) was performed for all five datasets and mean trait values, standard deviation (SD), and coefficient of variation (CV%) were determined with mixed models implemented in the lme4 package in R (Bates et al., 2015). For each single-environment dataset, the following mixed linear model was fitted: (Equation 1),

(1) Yij=μ+Gi+Rj+ϵij

where, Y*ij* represents the phenotypic response observed in the *j*^th^ replication for the *i*^th^ genotype, μ is the overall mean, G*i* is the effect of the *i*^th^ genotype, R*j* is the effect of the *j*^th^ replication, and ϵ*ij* represents the residual error term associated with the observation Y*ij*. Genotype and environment were considered as a random effect. The homogeneity of variance among the combined environments was tested using Levene’s test (Levene, 1960). Missing phenotypic observations, when present, were handled directly by restricted maximum likelihood estimation within the mixed-model framework; no phenotypic imputation was applied before analysis (Piepho et al., 2008).

For the All-combined dataset, the effects of genotype, environment, and genotype x environment interaction, and replication nested within environment were fitted using the following mixed model shown in Equation 2,

(2) Yijk=μ+Gi+(GE)ij+(ER)jk+ϵijk

where, Y*ijk* represents the phenotypic response observed the *i*^th^ genotype in the *j*^th^ environment and *k*^th^ replication, μ is the overall mean, G*i* is the random effect of the *i*^th^ genotype, ​(GE)*ij* is the random genotype x environment interaction, (ER)*jk* is the random effect of replication nested within environment, and ϵ*ijk* represents the residual error term associated with the observation Y*ijk*.

Pearson correlation coefficients were computed to evaluate both the strength and direction of relationships among the measured traits in the combined dataset, using the (psych) package in R (v4.5.1). Best linear unbiased predictions (BLUPs) were generated for all datasets, GAP-24/25, IHF-25, JPC-25, and the All-combined, using the (lme4) package in R (Bates, 2010). These estimates are referred to as BLUP_GAP-24/25, BLUP_IHF-25, BLUP_JPC-25, and BLUP_All, respectively. BLUPs were used to account for experimental design effects and environmental heterogeneity through mixed models and are more informative than raw arithmetic means when summarizing genotype performance across unbalanced or multi-environment data. Broad-sense heritability was calculated separately for each year’s dataset using the following Equation 3:

(3) $$H^{2}=\frac{\sigma_{G}^{2}}{\sigma_{G}^{2}+\sigma_{e}^{2}/n}$$

where $H^{2}$is the heritability estimate, $\sigma_{G}^{2}\;$represents the genetic variance, $\sigma_{e\;\;}^{2}$is the residual variance, and n is the number of replications.

Broad-sense heritability for the combined dataset was estimated using Equation 4:

(4) $$H^{2}=\frac{\sigma_{G}^{2}}{\sigma_{G}^{2}+\frac{\sigma_{GE}^{2}}{n_{E}}+\frac{\sigma_{ER}^{2}+\sigma_{e}^{2}}{n_{E}n_{R}}}$$

where, $\sigma_{GE}^{2}$ variation attributed to G x E interaction, $\sigma_{ER}^{2}$ refers to variation due to replications within each environment, and $n_{E}$ and $n_{R}$ are the harmonic mean numbers of environments and replications, respectively. Broad-sense H^2^ was reported because the objective was to quantify repeatability of whole-genotype performance in an association panel evaluated as entries in field trials, not to partition additive variance for a defined pedigree-based mating design.

### 2.4 Genotypic data analysis

#### 2.4.1 Genotyping, SNPs quality control, linkage disequilibrium and population structure

The SRWW diversity panel was genotyped at the USDA-ARS genotyping laboratory, Raleigh, North Carolina. DNA was extracted from freeze-dried leaf tissues and genotyping-by-sequencing (GBS) was performed using Illumina HiSeq 2500 after double digestion of genomic DNA with *Pstl* and *Msel* restriction enzymes, as described in Guo et al. (2020). SNP calling was carried out using TASSEL-GBS v5.2.49 discovery pipeline (Bradbury et al., 2007). Sequence reads from the Illumina platform were aligned to the IWGSC Chinese Spring RefSeq v1.0 assembly with the Burrows-Wheeler Aligner (v0.7.17-r1188) (Li and Durbin, 2010). Quality control prior to and after imputation included retaining only biallelic markers, removing SNPs with >50% missing data or minor allele frequency (MAF) <5%, and excluding genotypes with >85% missing data. Missing genotypes were imputed with Beagle 5.1, after which SNPs with MAF <5% or heterozygote frequency >10% were removed (Guo et al., 2020), to reduce potential false MTAs, while the remaining SNPs were utilized for subsequent analysis.

Linkage disequilibrium (LD) was estimated as the squared allele frequency (r^2^) between pairs of SNP markers as described by Hill and Weir (1988) using the TASSEL v 5.0 software package (Bradbury et al., 2007). Genome-wide and chromosome-wise LD-decay plots were generated, and the LD-decay threshold of (r^2^ ≥ 0.2) was used as the operational criterion for grouping closely linked significant SNPs into QTL regions because this threshold is widely used in wheat to approximate local LD support around associated loci. Population structure was assessed by principal component analysis (PCA) in genomic association and prediction integrated tool (GAPIT) package in R. The number of PCs retained as covariates in GWAS was chosen from the scree plot and by comparing model fit and p-value inflation in Q-Q plots; the final number of PCs used in the association models was 4.

#### 2.4.2 GWAS analysis

The SRWW panel comprising 182 genotypes was used for GWAS analysis to identify significant genomic regions associated with forage-quality traits. GWAS was conducted in GAPIT version 3 using four different models: namely the general linear model (GLM), mixed linear model (MLM), multiple loci mixed model (MLMM), and fixed and random model circulating probability unification (FarmCPU) (Wang and Zhang, 2021) package implemented in R v4.3.3. PCs were fitted as fixed covariates to account for population structure, and kinship was incorporated where appropriate through GAPIT model implementation to account for relatedness among lines. The number of significant SNPs and the quantile-quantile (Q-Q) plots observed by each model were evaluated to determine the most appropriate model for this study. FarmCPU was selected as the primary model because it provided the best compromise between statistical power and control of false positives for these data, which is consistent with its intended design and with recent wheat model-comparison studies (**Supplementary Figure 2**).

The Bonferroni-adjusted genome-wide threshold was considered overly conservative for this dataset because of the moderate panel size, strong marker correlation due to LD, and the risk of excessive type II error under a highly polygenic architecture. Therefore, a less stringent significance cutoff of 1×10^-4^ (Wang et al., 2016) was used for detecting significant MTAs, following precedent in crop GWAS that balance marker discovery with subsequent biological and LD-based filtering. False positives were further constrained by requiring acceptable Q-Q plot behavior, accounting for structure and relatedness in the model, and consolidating nearby significant SNPs into LD-supported QTL regions rather than interpreting isolated marker signals independently. For each significant marker, the proportion of phenotypic variance explained (PV) was estimated from regression of the phenotype on the associated marker. Significant MTAs on the same chromosome were grouped into a single QTL when they were in LD at or above the study LD threshold (r^2^ ≥ 0.2) and fell within the same local support interval; otherwise, they were considered distinct loci. Stable loci were defined as QTL detected in at least two datasets among BLUP_GAP-24/25, BLUP_IHF-25, BLUP_JPC-25, and BLUP_All. QTLs explaining >10% of the PV were classified as high-confidence, major-effect QTLs for downstream interpretation and candidate-gene analysis. Because low H^2^ reduces GWAS detection power, the combined interpretation of single-environment, multi-environment, and stable loci was used to prioritize robust associations. QTL nomenclature followed the updated wheat nomenclature guidelines proposed by Boden et al. (2023).

#### 2.4.3 Favorable allele and additive allele effect of major effect QTLs

Allelic effects were examined for all major-effect QTLs identified in this research study. To assess these effects, the SRWW panel was divided into three genotype classes based on the allelic state of each associated marker, homozygous for the favorable allele, heterozygous, and homozygous for the unfavorable allele. This classification was determined by the direction of the marker’s effect on trait performance. Favorable alleles were defined as those associated with improved forage-quality characteristics in SRWW, including increased DM, CP, TDN, SUG, and RFQ, along with lower values of ADF, NDF, and ADL. To statistically assess differences among genotype classes, Tukey’s Honestly Significant Difference (HSD) test was performed using BLUP values for the associated traits derived from multiple datasets.

The effects of favorable allele accumulation were further evaluated for traits controlled by multiple major-effect QTLs. For each QTL, the most strongly associated marker was selected as a representative, and genotypes were subsequently grouped according to the total number of favorable alleles carried, including a baseline class consisting of lines lacking favorable alleles. Genotypes within each allele class were identified, and their trait performance was quantified using BLUPs derived from the All-combined dataset. Differences among allele classes were statistically assessed using Tukey’s HSD test.

#### 2.4.4 Candidate gene discovery and novelty testing

Candidate gene identification was performed by examining genes located in close physical position of 5kb window size to the significant MTAs. The Chinese Spring RefSeq v1.1 reference genome assembly (Appels et al., 2018), accessed through Ensembl Plants (http://plants.ensembl.org/), was used to retrieve gene models underlying the identified QTL regions. Functional characterization of the candidate genes was conducted using multiple annotation resources, including the InterPro database (https://www.ebi.ac.uk/interpro/search/sequence/), the Wheat Expression Browser (http://www.wheat-expression.com/), and Wheatomics 1.0 (Ma et al., 2021). Candidate-gene prioritization considered both annotated molecular function and expression evidence in tissues or conditions relevant to plant development, metabolism, or stress response. To assess the novelty of the detected QTLs, the physical positions of the identified loci were compared with previously reported QTLs and MTAs in wheat and related cereals using the Chinese Spring reference in publicly available databases, including NCBI (https://www.ncbi.nlm.nih.gov/) and PlantBioinfoPF (https://urgi.versailles.inra.fr/; Alaux et al., 2018). A QTL was considered previously reported when the confidence interval or LD-supported interval of the present locus overlapped the physical interval of an earlier published QTL/MTA for the same or a closely related trait; otherwise, it was classified as putatively novel.

## 3 Results

### 3.1 Phenotyping of the diversity panel

Single location ANOVA revealed significant variation (p<0.001 to p<0.05) among the genotypes for all forage-quality traits except DM and SUG for GAP-24, RFQ for GAP-25 and DM and ADL for IHF-25 (**Supplementary Table 1**). However, strong environmental effects were observed at the JPC-25 location and ANOVA revealed non-significant differences among genotypes for all quality traits. The combined ANOVA also showed significant variation (p<0.001 to p<0.05) among genotypes for most forage quality traits except for DM, ADL, TDN, and RFQ (**Supplementary Table 1**). DM ranged from 11.35 to 34.67%, with a mean value of 19.24% (**Table 1**). The IHF-25 location had a higher mean value (22.17%) for DM than GAP-25 (20.79%), GAP-24 (17.80%), and JPC-25 (16.19%) (**Supplementary Table 1**). CP also showed variable values ranging from 10.40% to 32.24%, with a mean value of 19.58%. For fiber components, the ADF and NDF ranges were 13.21% to 33.38% and 35.43% to 62.88%, respectively, with mean values of 21.99% and 46.56% (**Supplementary Table 1**). Interestingly, JPC-25 location had slightly higher ADF and NDF mean values (23.63% and 49.53%, respectively) than GAP-24/25 and IHF-25 (**Supplementary Table 1**). ADL had an overall mean value of 2.54%, ranging from 0.68% to 6.88%, with a higher mean at GAP-24 (5.07%). TDN and SUG had mean values of 67.54% and 13.09%, ranging from 57.33% to 74.68% and 8.42% to 19.31%, respectively, with GAP-25 showing the higher mean percentages (71.65% and 15.85%, respectively) (**Supplementary Table 1**; **Table 1**). RFQ also showed wide variation, ranging from 47.01 to 219.89, with an overall mean value of 159.47 (**Table 1**). As for the majority of traits, GAP-25 location showed a higher value (196.28) for RFQ than IHF-25 (176.01), GAP-24 (139.42), and JPC-25 (126.17) (**Supplementary Table 1**). CV ranged from 2.02% for TDN to 12.39% for CP, with relatively higher CV observed for CP (12.39%), ADL (11.57%), RFQ (11.09%), and DM (9.45%), indicating considerable variation for these traits within the test location (**Table 1**). Large variation observed among genotypes is usually favorable for breeding because it increases the opportunity to identify parents that combine high CP and RFQ with reduced fiber fractions, a pattern also emphasized in previous winter wheat forage studies (Kim and Anderson, 2015; Maulana et al., 2019; West et al., 2024).

**Table 1:** Descriptive statistics and heritability (H2) estimation for 8 forage-quality traits studied in 182 SRWW lines.

Trait^a^	General statistics of the population						
	Mean	SD^b^	CV^c^	Min^d^	Max^e^	SE^f^	H^2 g^
DM	19.24%	1.82	9.45	11.35	34.67	0.048	0.00052
CP	19.58%	2.43	12.39	10.40	32.24	0.064	0.08563
ADF	21.99%	1.89	8.60	13.21	33.38	0.05	0.24306
NDF	46.56%	2.60	5.59	35.43	62.88	0.068	0.325
ADL	2.54%	0.29	11.57	0.68	6.88	0.008	0.00352
TDN	67.54%	1.36	2.02	57.33	74.68	0.036	0.02422
SUG	13.09%	0.97	7.41	8.42	19.31	0.025	0.162
RFQ	159.47	17.69	11.09	47.01	219.89	0.464	0.00433

^a^ Forage-quality traits DM, dry matter; CP, crude protein; ADF, acid detergent fiber; NDF, neutral detergent fiber; ADL, acid detergent lignin; TDN, total digestible nutrients; SUG, sugar content; RFQ, relative forage quality.

^b^ SD, standard deviation from the All-Combined dataset.

^c^ CV, coefficient of variation from the All-Combined dataset.

^d^ Minimum value obtained for traits from the All-Combined dataset.

^e^ Maximum value obtained for traits from the All-Combined dataset.

^f^ SE, standard error of mean.

^g^ Broad-sense heritability estimates for the traits from the All-Combined dataset.

Broad sense heritability (H^2^) estimates for the forage-quality traits showed substantial variability across traits (**Table 1**). H^2^ values ranged from 0.001 to 0.325 in the All-combined dataset, indicating generally low to moderate genetic control of the studied traits. Among the measured traits, NDF exhibited the highest heritability (H² = 0.325), suggesting a moderate proportion of phenotypic variation attributable to genetic control. ADF and SUG showed lower H^2^ levels, with H² of 0.243 and 0.162, respectively. In contrast, several other forage-quality traits exhibited very low H^2^, including CP (H² = 0.086), TDN (H² = 0.024), RFQ (H² = 0.004), ADL (H² = 0.004), and DM (H² = 0.001). While H^2^ values for single-location datasets ranged from 0.114 to 0.350 (GAP-24), with H^2^ of 0.114 for DM and H^2^ of 0.350 for CP (**Supplementary Table 1**), GAP-25 H^2^ ranged from 0.244 to 0.755 for RFQ and NDF, respectively. H^2^ values for IHF-25 ranged from 0.079 to 0.676 for DM and ADF (**Supplementary Table 1**).

**Figure 1:**
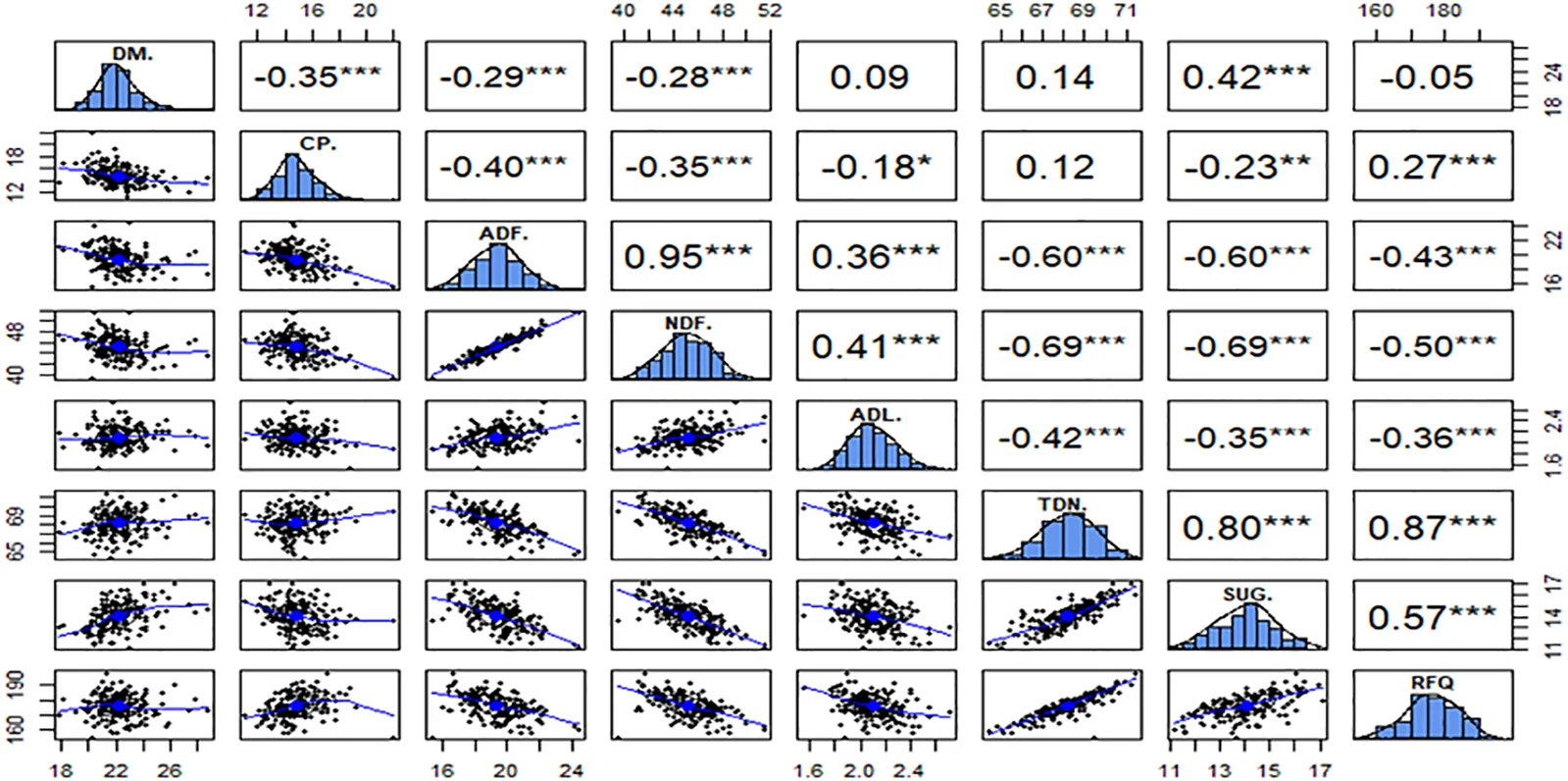
Correlation analysis of eight forage-quality traits among 182 SRWW lines. Combined data from four environments across two years (2023–2025, All-Combined) were used for the correlation analysis. DM, dry matter; CP, crude protein; ADF, acid detergent fiber; NDF, neutral detergent fiber; ADL, acid detergent lignin; TDN, total digestible nutrients; SUG, sugar; RFQ, relative forage quality. *** significant at p<0.001, ** significant at p<0.01, * significant at p<0.05 probability level.

The correlation analysis revealed several significant relationships among forage-quality traits evaluated in this study (**Figure 1**). DM exhibited negative correlations with most quality traits, including CP (r = -0.35), ADF (r = -0.29), and NDF (r = -0.28). While DM showed a positive association with SUG (r = 0.42). CP displayed negative correlations with ADF (r = -0.40), NDF (r = -0.35), SUG (r = -0.23), and ADL (r = -0.18), but demonstrated positive correlations with RFQ (r = 0.27). The fiber fractions showed strong internal relationships. ADF had a positive correlation with NDF (r = 0.95) and ADL (r = 0.36). As expected, ADF, and NDF exhibited pronounced negative correlations with TDN and SUG (r = -0.60 and r = -0.69, respectively), and RFQ (r = -0.43 and -0.50, respectively). In the case of ADL, it also showed a negative association with TDN, SUG, and RFQ (r = -0.42, -0.35, and -0.36, respectively). Among the quality-enhancing traits, TDN showed positive correlations with both SUG (r = 0.80) and RFQ (r = 0.87), while SUG was also highly correlated with RFQ (r = 0.57) (**Figure 1**).

### 3.2 Population structure, marker distribution, and LD

**Figure 2:**
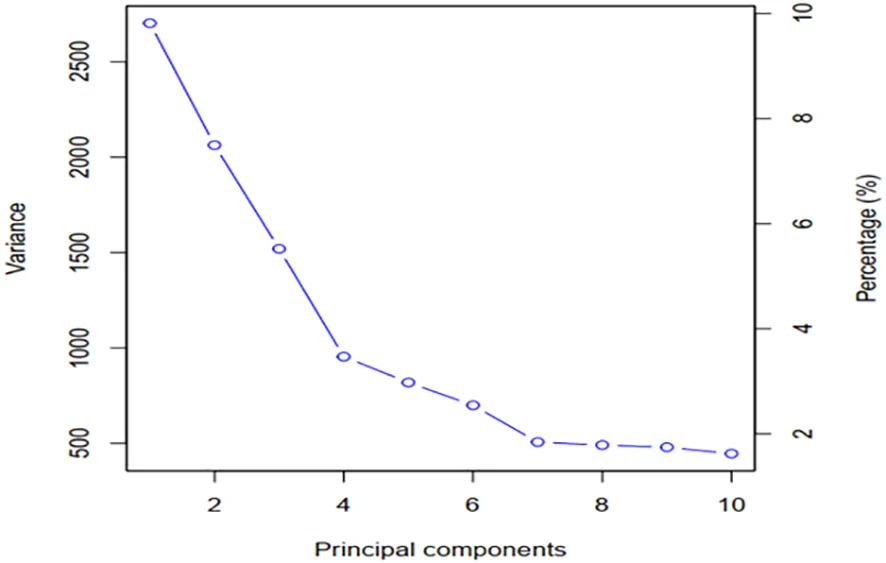
Percentage of variance explained by the first ten principal components (PCs).

Population structure was assessed using PCs based on the genome-wide SNP data. The scree plot of the first 10 PCs showed a steep decline in variance explained by the first 4 components, after which the curve gradually leveled off (**Figure 2**), indicating that these components capture the major population structure in the panel. These four PCs were included as covariates in GWAS. A total of 47,339 high-confidence SNPs markers were retained after quality filtration. Among the three subgenomes, B subgenome exhibited the highest SNP density, comprising 23,679 markers (50%), followed by the A subgenome (17,055, 36%), while the D subgenome had the least (6,604, 14%). Variation in marker density among subgenomes likely influenced the observed distribution of MTAs, with the higher number detected on the B subgenome reflecting its greater marker coverage and, consequently, an increased chance of detecting linkage with causal variants. LD decay analysis across the whole genome revealed a half-decay distance of approximately 6.36 Mb (**Supplementary Figure 1**). Individually, the A subgenome showed the most rapid LD half-decay distance at 2.80 Mb, followed by the D subgenome at 5.13 Mb, and the B subgenome exhibited the slowest LD half-decay distance at 10 Mb. Chromosomal LD decay values varied considerably, with the shortest decay observed on chromosome 4D (41 bp) and the longest on chromosome 1D (43 Mb) (**Supplementary Figure 1**). The observed whole-genome half-decay distance reflects moderate mapping resolution in this elite wheat breeding panel. Consistent with prior reports for advanced germplasm, slower LD decay occurs due to shared ancestry and historical selection, which maintain longer haplotype blocks (Breseghello and Sorrells, 2006). Accordingly, the present panel is suitable for genome-wide detection of associated regions, but the multi-megabase LD extent limits precise positional resolution for causal gene identification in some chromosomal regions. An r² cutoff of 0.2 served as the threshold for defining LD among marker pairs, and any MTA on the same chromosome with pairwise value ≥ 0.2 was considered as linked and grouped into the same putative QTL. The use of r² = 0.2 follows common practice in association mapping because it approximates the point at which LD has decayed to background levels while remaining stringent enough to avoid fragmenting a single association peak into multiple artificial QTL.

### 3.3 MTA’s identification for forage-quality traits

Among all environments, the JPC-25 location had the greatest environmental effect, and ANOVA revealed non-significant differences between genotypes for all forage-quality traits. So, there was no need to perform GWAS analysis for JPC-25. Among the four tested models (GLM, MLM, MLMM, and FarmCPU), we found FarmCPU to have better control over false positive and false negative associations. Thus, FarmCPU was used to run GWAS for all forage-quality traits studied (**Supplementary Figure 2**). A total of 282 MTAs were identified (P < 1×10^-4^) from all four datasets (**Supplementary Table 2**). Of the 282 MTAs, 77 were associated with DM, 72 for CP, 49 for RFQ, 40 for SUG. There were 17 for ADL, whereas ADF had 11 MTAs. NDF and TDN each had 8 MTAs, respectively. Thus, DM and CP were the traits with the highest number of MTAs, and NDF and TDN with the lowest number of MTAs (**Supplementary Table 2**).

MTAs were discovered across 19 wheat chromosomes, excluding 1D and 6A (**Supplementary Table 2**). Chromosome 2D and 2B exhibited the highest number of MTAs, totaling 55 and 47, respectively, while chromosomes 3D, 4D, 5D, and 7D each had only one MTA. The B subgenome featured the greatest number of MTAs (122), followed by the D subgenome (85) and A subgenome (75) (**Supplementary Table 2**).

The marker S3D_555274375 contributed to the highest percentage of PV explained for DM. Notably, this marker exhibited the highest PV among all tested forage-quality traits, contributing to 17.3% (**Supplementary Table 2**). Similarly, markers S2D_9928132 and S3A_577990641 accounted for the highest PV for CP, at 15.1% and 15.4%, respectively. Interestingly, out of 72 MTAs for CP, 55 were identified on chromosome 2D. For fiber fractions, ADF and NDF, the markers S2A_711828719 and S1B_16990314 explained the highest PV of 10.3% and 9.5%, respectively. For RFQ, two markers S1B_151287916 and S1B_151287920 were associated with the highest PV, explaining 11.6%. Additionally, of the 49 MTAs related to RFQ, 45 were on the B chromosomes (**Supplementary Table 2**). For SUG, the marker S4B_670800146 accounted for the highest PV of 11.0% along with the markers S7A_2974209, S7A_3426596, S7A_3426616, S7A_3426620, and S7A_3426628 associated with SUG, explaining 10.6% of PV. All 40 MTAs for SUG were identified on the A and B subgenomes (**Supplementary Table 2**). In the case of ADL, it was associated with the markers S6B_6242037 and S6B_6242055 explaining the highest PV of 13.0% for both. Finally, the marker associated with TDN S5A_19997583 accounted for the highest PV, at 8.3%.

### 3.4 Major effect QTLs for forage-quality traits

Based on the specific LD critical value of 0.2, we identified 282 significant MTAs and resolved them into 121 distinct QTLs (**Supplementary Table 2**). Furthermore, we identified 27 QTLs explaining ≥ 10% of PV, which were considered major-effect QTLs, with no major-effect QTLs identified for NDF and TDN (**Table 2**). Under the working definition used in this study, loci explaining ≥ 10% of PV were classified as major-effect because they exceeded the small-effect background expected for most complex forage-quality traits in wheat, However, these loci should be interpreted as putative moderate-to-large effect regions pending validation in independent populations and environments, particularly because effect sizes estimated in discovery panels of moderate size can be upwardly biased (Xu, 2003). DM was controlled by seven major-effect QTLs, identified on chromosome 1A, designated *QDm.uga-1A.1* through *QDm.uga-1A.7*, each explaining approximately 11-12% PV, detected particularly in the BLUP_GAP-25 and BLUP_IHF-25 datasets. Among these, multiple QTLs were represented by two or more markers in strong LD, confirming the presence of distinct genomic regions influencing DM (**Supplementary Table 2**). Additionally, a major-effect QTL, *QDm.uga-3A*, for DM explaining 11.0% PV was located on chromosome 3A. Another major-effect QTL on chromosome 3D, detected in the BLUP_GAP-24 dataset, *QDm.uga-3D*, exhibited the largest effect on DM, explaining up to 17.3% PV, making it one of the most influential loci detected for DM (**Table 2**).

**Table 2:** Major-effect QTLs for forage-quality traits from four BLUP datasets, BLUP-GAP-24/25, BLUP-IHF-25, and BLUP-All.

QTL^a^	SNP^b^	Trait^c^	Dataset^d^	Allele^e^	MAF^f^	P.value^g^	Effect^h^	PV^i^	Candidate gene-ID^j^	Associated protein^k^
*QDm.uga-1A.1*	S1A_303679238	DM	BLUP_GAP-25	G/A	0.058	2.52786E-05	0.43	**11.02**	**-**	**-**
*QDm.uga-1A.2*	S1A_297738248	DM	BLUP_GAP-25	G/T	0.064	8.2428E-05	0.39	**10.99**	**-**	**-**
*QDm.uga-1A.3*	S1A_299486443	DM	BLUP_GAP-25	C/T	0.061	3.86547E-05	0.41	**12.09**	**-**	**-**
*QDm.uga-1A.4*	S1A_302922335	DM	BLUP_GAP-25	G/C	0.061	3.86547E-05	0.41	**10.99**	**-**	**-**
*QDm.uga-1A.5*	S1A_305110930	DM	BLUP_GAP-25	G/A	0.061	3.86547E-05	0.41	**11.02**	**-**	**-**
*QDm.uga-1A.6*	S1A_180001065	DM	BLUP_GAP-25	G/A	0.052	4.52376E-05	0.43	**11.55**	**-**	**-**
*QDm.uga-1A.7*	S1A_162319212	DM	BLUP_IHF-25	C/G	0.081	2.7347E-05	-0.11	**12.36**	**-**	**-**
*QRfq.uga-1B*	S1B_151287916	RFQ	BLUP_GAP-24	G/A	0.090	3.0266E-05	3.45	**11.55**	**-**	**-**
*QAdf.uga-1B*	S1B_126990314	ADF	BLUP_IHF-25	C/T	0.157	2.1447E-05	-0.55	**10.11**	**-**	**-**
*QAdf/Adl.uga-2A*	S2A_711828719	ADF	BLUP_GAP-24	G/A	0.346	1.5539E-05	-0.19	**10.31**	**-**	**-**
	S2A_711526601	ADL	BLUP_GAP-24	G/T	0.381	2.39817E-05	-0.03	**11.30**	**-**	**-**
*QCp.uga-2D*	S2D_9928132	CP	BLUP_IHF-25	C/G	0.104	3.9575E-06	-0.36	**15.10**	**-**	**-**
*QDm.uga-3A*	S3A_724527135	DM	BLUP_GAP-25	G/A	0.038	1.89042E-05	0.76	**11.00**	**-**	**-**
*QCp.uga-3A*	S3A_577990641	CP	BLUP_IHF-25	G/A	0.128	3.4601E-05	-0.26	**15.42**	**-**	**-**
*QRfq.uga-3B.1*	S3B_566641307	RFQ	BLUP_GAP-25	C/T	0.134	3.8074E-05	0.43	**10.72**	TraesCS3B02G356400	Transcriptional control or nucleic acid binding
		RFQ	BLUP_All	C/T	0.041	5.50381E-05	0.08	7.65	–	–
*QRfq.uga-3B.2*	S3B_570524495	RFQ	BLUP_GAP-25 BLUP_All	G/A	0.041 0.041	3.8074E-05 5.50381E-05	1.77 0.08	**10.72** 7.65	**-**	**-**
*QDm.uga-3D*	S3D_555274375	DM	BLUP_GAP-24	T/A	0.003	1.1407E-12	-0.81	**17.29**	**-**	**-**
*QCp.uga-4A.1*	S4A_712451436	CP	BLUP_IHF-25	G/A	0.049	7.3492E-05	-0.39	**12.67**	*TraesCS4A02G443700*	Cytochrome P450, monooxygenase activity, iron ion binding
*QRfq.uga-4A*	S4A_725532798	RFQ	BLUP_GAP-25	C/T	0.224	3.84041E-05	0.99	**11.23**	**-**	**-**
*QCp.uga-4A.2*	S4A_405677816	CP	BLUP_GAP-24	G/C	0.073	9.7237E-06	-0.58	**11.35**	**-**	**-**
*QCp.uga-4A.3*	S4A_12623190	CP	BLUP_IHF-25	A/G	0.026	8.12723E-05	-0.52	**13.67**	*TraesCS4A02G019500*	Pentatricopeptide repeats (PPR) protein, translation in mitochondria and/or chloroplasts
*QSug.uga-4B*	S4B_670800146	SUG	BLUP_GAP-25	T/C	0.323	5.6887E-05	0.22	**11.04**	*TraesCS4B02G396400*	UDP-Glycosyltransferase/glycogen phosphorylase activity
*QAdl.uga-5A*	S5A_682890015	ADL	BLUP_GAP-25	C/T	0.142	3.5098E-05	0.04	**11.22**	**-**	**-**
*QAdl.uga-5B.1*	S5B_605517698	ADL	BLUP_IHF-25	A/G	0.113	4.7201E-05	0.02	**11.54**	TraesCS5B02G430200	A20/AN1-type zinc finger protein, stress-associated protein (SAP)
*QAdl.uga-5B.2*	S5B_486483108	ADL	BLUP_GAP-25	T/C	0.483	8.7536E-05	0.03	**10.53**	*TraesCS5B02G302000*	RNA recognition motif-containing protein
*QAdl.uga-5B.3*	S5B_607020013	ADL	BLUP_IHF-25	G/A	0.110	4.4172E-05	0.02	**11.32**	**-**	**-**
*QAdl.uga-6B*	S6B_6242037	ADL	BLUP_GAP-25	C/T	0.041	2.9139E-05	0.08	**12.97**	**-**	**-**
*QDm/Sug.uga-7A*	S7A_3765553	DM	BLUP_GAP-24	C/T	0.093	2.0572E-14	-0.11	6.40	–	–
	S7A_2974209	SUG	BLUP_GAP-25	T/C	0.157	3.9191E-05	0.27	**10.64**	**-**	**-**

^a^ Major-effect QTL.

^b^ SNP associated with major-effect QTL.

^c^ Forage-quality traits. DM, dry matter; CP, crude protein; ADF, acid detergent fiber; NDF, neutral detergent fiber; ADL, acid detergent lignin; TDN, total digestible nutrients; SUG, sugar; RFQ, relative forage quality.

^d^ BLUP datasets used for identification of the marker-trait association. BLUP-GAP-24, BLUP values from Plains 2024 dataset; BLUP-GAP-25, BLUP values from Plains 2025 dataset; BLUP-IHF-25, BLUP values from Iron Horse Farm 2025 dataset; BLUP-All, BLUP values from All-combined dataset.

^e^ Allele combination for the locus.

^f^ MAF, minor allele frequency.

^g^ P.value.

^h^ Allelic effect on the trait. In the GAPIT-based GWAS result, the effect is estimated for the second marker in alphabetical order which means it could be a minor or major allele effect.

^i^ Percentage of phenotypic variance (PV) explained by the marker.

^j^ Candidate genes identified for the QTL.

^k^ Protein produced by the candidate gene.

Five major-effect QTLs for CP were identified on chromosomes 2D (one), 3A (one), and 4A (three). Notably, *QCp.uga-2D* and *QCp.uga-3A* explained 15.1% and 15.4% PV, respectively, indicating strong effects on protein accumulation. Three additional QTLs on chromosome 4A (*QCp.uga-4A.1, QCp.uga-4A.2*, and *QCp.uga-4A.3*) each contributing 11 to 13% PV across datasets, BLUP_IHF-25 and BLUP_GAP-24 (**Table 2**). Among all these, QTL *QCp.uga-2D* and *QCp.uga-4A.1* were represented by three and nine markers in strong LD (**Supplementary Table 2**). For SUG, there were two major-effect QTLs *QSug.uga-4B*, located on chromosome 4B, identified in the BLUP_GAP-25 dataset, explained 11.0% PV, while another important locus, *QDm/Sug.uga-7A*, exhibited a multi-trait association, with co-located effects on both traits, contributing up to 10.6% PV for SUG explaining 6.4% PV for DM (**Table 2**). Emphasize that the DM effect should be interpreted in the context of its correlation with SUG (r = 0.42) and across multiple environments.

For ADF, two QTLs *QAdf.uga-1B* (BLUP_IHF-25, 10.1% PV) and *QAdf/Adl.uga-2A* (BLUP_GAP-24, 10.3% PV) were identified. The latter has also influenced ADL and explained up to 11.3% PV, reflecting a co-localized multi-trait QTL within the same genomic region (**Table 2**). Additionally, five major-effect QTLs for ADL were identified, located on chromosomes 5A (1), 5B (3), and 6B (1). Among these, *QAdl.uga-6B* (BLUP_GAP-25) showed a strong effect, explaining 13.0% PV, while *QAdl.uga-5A* (BLUP_GAP-25) contributed 11.2% PV of ADL. A QTL cluster on chromosome 5B (*QAdl.uga-5B.1*, *QAdl.uga-5B*.2, and *QAdl.uga-5B*.3) was identified across BLUP_GAP-25 and BLUP_IHF-25, each explaining ~11–12% PV, marking this a key region regulating lignin content (**Table 2**).

Four major-effect QTLs were identified for RFQ. These QTLs were distributed across chromosomes 1B (1), 3B (2), and 4A (1). The QTL *QRfq.uga-1B* (from BLUP_GAP-24), explained 11.6% PV and represented the strongest RFQ QTL detected. Two additional QTLs on chromosome 3B (*QRfq.uga-3B.1* and *QRfq.uga-3B.2*) each explained 10.7% PV: these two QTLs, *QRfq.uga-3B.1* and *QRfq.uga-3B.2* were consistently identified across multiple datasets (BLUP_GAP-25 and BLUP_All), indicating stable expression. Additionally, the QTL on chromosome 4A (*QRfq.uga-4A* from BLUP_GAP-25 dataset) contributed 11.2% PV (**Table 2**).

### 3.5 Allelic effect of major-effect QTLs

Allelic effects were evaluated for all major-effect QTLs detected (**Table 3**). A clear difference was observed between genotypes carrying the favorable and unfavorable alleles for nearly all major-effect QTLs. Alleles associated with improved performance, including increased DM, CP, RFQ, and SUG, or reduced ADF and ADL, were interpreted as favorable. A cluster of major-effect QTLs on chromosome 1A showed largely consistent allelic effects on DM. At *QDm.uga-1A.1* and *QDm.uga-1A.2* (*S1A_303679238* and *S1A_297738248*) for DM, the lines with the homozygous favorable allele “G” (GG, 169 and 167 lines) had 0.79% higher DM than lines carrying the homozygous unfavorable allele “A” (AA, 12 lines) (**Table 3**). QTLs *QDm.uga-1A.4* and *QDm.uga-1A.5* had similar patterns for DM, where the lines with homozygous favorable allele “G” (169 and 168 lines) produced 0.79% higher DM than their homozygous unfavorable alleles (CC and AA). Similarly, for *QDm.uga-1A.3* associated with DM (*S1A_299486443*), the difference between the homozygous favorable (CC, 161 lines) and unfavorable allele (TT, 10 lines) was 0.85% (**Table 3**). For *QDm.uga-1A.6* and *QDm.uga-1A.7* identified for DM, the lines carrying the favorable homozygous “G” allele (172 and 5 lines) consistently showed DM values 0.89% and 0.27% higher than those homozygous for the unfavorable alleles (AA and CC). Overall, loci on chromosome 1A indicated that G allele at these contributed positively to DM, with unfavorable alleles typically reducing DM by nearly one percent (**Table 3**).

**Table 3:** Major QTLs, SNPs, forage-quality traits, alleles, and allelic effects of significant markers associated with major-effect QTLs.

QTL^a^	SNP^b^	Trait^c^	MAF^d^	Genotype^e^	N^f^	Mean BLUP^g^	TUKEY HSD test^h^
*QDm.uga-1A.1*	S1A_303679238	DM	0.058	AA	12	20.05	A
				AG	1	20.69	AB
				**GG**	169	20.84	B
*QDm.uga-1A.2*	S1A_297738248	DM	0.064	**GG**	167	20.84	B
				GT	3	20.88	AB
				TT	12	20.05	A
*QDm.uga-1A.3*	S1A_299486443	DM	0.061	**CC**	161	20.84	B
				CT	1	20.82	AB
				TT	10	19.99	A
*QDm.uga-1A.4*	S1A_302922335	DM	0.061	CC	12	20.05	A
				GC	1	20.82	AB
				**GG**	169	20.84	B
*QDm.uga-1A.5*	S1A_305110930	DM	0.061	AA	12	20.05	A
				AG	2	20.76	AB
				**GG**	168	20.84	B
*QDm.uga-1A.6*	S1A_180001065	DM	0.052	AA	10	19.95	A
				**GG**	172	20.84	B
				AG	0	NA	–
*QDm.uga-1A.7*	S1A_162319212	DM	0.081	CC	149	21.67	A
				CG	18	21.78	B
				**GG**	5	21.94	B
*QRfq.uga-1B*	S1B_151287916	RFQ	0.090	AA	9	132.05	A
				GA	13	137.41	B
				**GG**	150	139.96	B
*QAdf.uga-1B*	S1B_126990314	ADF	0.157	**CC**	152	18.73	A
				CT	6	19.19	AB
				TT	24	19.74	B
*QAdf/Adl.uga-2A*	S2A_711828719	ADF	0.346	AA	58	26.73	B
				GA	3	26.67	AB
				**GG**	111	26.38	A
	S2A_711526601	ADL	0.381	**GG**	104	5.04	A
				GT	5	5.08	AB
				TT	63	5.11	B
*QCp.uga-2D*	S2D_9928132	CP	0.104	CC	162	14.28	A
				CG	4	14.89	B
				**GG**	16	14.97	B
*QDm.uga-3A*	S3A_724527135	DM	0.038	GA	13	20.13	A
				**GG**	159	20.84	B
				AA	0	NA	–
*QCp.uga-3A*	S3A_577990641	CP	0.128	**AA**	23	14.91	B
				GA	1	14.05	AB
				GG	158	14.28	A
*QRfq.uga-3B.1*	S3B_566641307	RFQ	0.134	**CC**	175	195.91	B
				TT	7	192.39	A
				CT	0	NA	–
		RFQ	0.041	**CC**	175	0.01	B
				TT	7	-0.14	A
				CT	0	NA	–
*QRfq.uga-3B.2*	S3B_570524495	RFQ	0.041	AA	7	192.39	A
				**GG**	175	195.91	B
				AG	0	NA	–
*QCp.uga-4A.1*	S4A_712451436	CP	0.049	**AA**	8	15.22	B
				GA	1	14.76	AB
				GG	163	14.31	A
*QRfq.uga-4A*	S4A_725532798	RFQ	0.224	**CC**	141	196.13	B
				CT	2	196.62	AB
				TT	39	194.45	A
*QCp.uga-4A.2*	S4A_405677816	CP	0.073	**CC**	10	20.25	B
				CG	7	19.25	AB
				GG	165	19.12	A
*QCp.uga-4A.3*	S4A_12623190	CP	0.026	AA	176	14.32	A
				AG	2	14.49	A
				**GG**	4	15.68	B
*QSug.uga-4B*	S4B_670800146	SUG	0.326	CC	54	15.12	A
				TC	3	15.28	AB
				**TT**	115	15.53	B
*QAdl.uga-5A*	S5A_682890015	ADL	0.142	CC	157	0.91	B
				CT	1	0.93	AB
				**TT**	24	0.84	A
*QAdl.uga-5B.1*	S5B_605517698	ADL	0.113	AA	152	1.67	B
				AG	1	1.64	AB
				**GG**	19	1.62	A
*QAdl.uga-5B.2*	S5B_486483108	ADL	0.483	CC	92	0.93	B
				CT	1	0.93	AB
				**TT**	89	0.88	A
*QAdl.uga-5B.3*	S5B_607020013	ADL	0.110	**AA**	19	1.62	A
				GG	153	1.67	B
				AG	0	NA	–
*QAdl.uga-6B*	S6B_6242037	ADL	0.041	CC	160	0.91	C
				CT	10	0.84	B
				**TT**	2	0.71	A
*QDm/Sug.uga-7A*	S7A_3765553	DM	0.093	CC	162	17.73	A
				CT	1	17.84	AB
				**TT**	19	17.88	B
	S7A_2974209	SUG	0.157	CC	26	14.96	A
				TC	2	15.19	AB
				**TT**	144	15.48	B

^a^ Major-effect QTL.

^b^ SNP associated with major-effect QTL.

^c^ Forage-quality traits. DM., dry matter; CP., crude protein; ADF., acid detergent fiber; NDF., neutral detergent fiber; ADL., acid detergent lignin; TDN., total digestible nutrients; SUG., sugar; RFQ, relative forage quality.

^d^ MAF, minor allele frequency.

^e^ Homozygous and heterozygous genotypes for each SNP. The allele in bold is the favorable allele for the trait.

^f^ N, number of wheat lines having respective genotype class for each marker.

^g^ Mean value of traits in BLUP_GAP-24/25, BLUP_IHF-25, and BLUP-All.

^h^ Tukey’s HSD test results where different letters represent significant differences at p<0.05 for each QTL.

Allelic differences for DM production were also detected on chromosomes 3A and 3D. At *QDm.uga-3A*, favorable allele “G” (159 lines) produced 0.71% higher DM than heterozygous GA lines, although no lines possessed the homozygous genotype AA (**Table 3**). Another multi-trait QTL, *QDm/Sug.uga-7A*, also influenced DM to a lesser degree (S7A_3765553) and SUG (S7A_2974209) had a favorable allele “T” for both DM and SUG, where the homozygous state of favorable allele (TT, 19 lines) had 0.15% higher DM and 0.52% of SUG (144 line) in comparison to the homozygous state of unfavorable allele (CC, 162 and 26 lines) (**Table 3**). Another QTL for SUG, *QSug.uga-4B* (*S4B_670800146*), where the favorable allele “T” (115 lines) is involved in increasing 0.41% of SUG (**Table 3**).

There was also a clear allelic difference observed at major-effect QTLs associated with RFQ identified on chromosomes 1B, 3B, and 4A. The QTL *QRfq.uga-1B* (*S1B_151287916*), exhibited the largest allelic effect for RFQ, where “G” was the favorable homozygous state allele with 150 lines showing 7.91 index value higher RFQ than the unfavorable homozygous state (AA, 9 lines), indicating G as the favorable allele. Two linked regions on chromosome 3B where *QRfq.uga-3B.1* and *QRfq.uga-3B.2* exhibited similar trends (**Table 3**). At both QTLs, CC and GG (175 lines each) produced 3.52 higher RFQ values than their alternative homozygous unfavorable alleles (TT and AA). These regions appear to represent a shared underlying QTL with a consistent positive effect on forage quality. At *QRfq.uga-4A* (*S4A_725532798*), lines carrying homozygous favorable allele “C” (141 lines) exhibited 1.68 higher RFQ values than unfavorable homozygous allele TT (39 lines) (**Table 3**).

For *QCp.uga-2D* associated with CP (*2D_9928132*), the lines with homozygous favorable allele “G” (GG, 16 lines) had recorded 0.69% higher CP than the lines with unfavorable homozygous allele (CC, 162 lines) (**Table 3**). At *QCp.uga-3A* (*S3A_577990641*), the homozygous favorable (AA, 23 lines) had 0.63% higher CP observed than 158 lines carrying unfavorable allele “G”. Thus, A was considered the favorable allele on chromosome 3A for CP. Similarly, three other QTLs on chromosome 4A also contributed significantly to CP expression. The QTL *QCp.uga-4A.1* (*S4A_712451436*), where the homozygous favorable allele “A” (AA, 8 lines) showed 0.91% higher CP in comparison to the homozygous unfavorable allele (GG, 163 lines) (**Table 3**). At *QCp.uga-4A.2* (*S4A_405677816*), the “C” allele (CC, 10 lines) was identified as the favorable allele and was associated with a 1.13% higher CP compared with lines carrying the unfavorable allele (GG, 165 lines). In contrast, *QCp.uga-4A.3* (*S4A_12623190*) favored the “G” allele (4 lines), as GG lines produced 1.36% higher CP than their alternative unfavorable homozygous lines (AA, 176 lines). This locus showed one of the largest allelic effects observed for CP. Overall, favorable alleles for CP varied by locus, with some QTLs showing substantial effect sizes that could be valuable for selection for high CP (**Table 3**).

For ADF, at major-effect QTL *QAdf.uga-1B*, associated with the marker *S1B_126990314* (**Table 3**). The lines homozygous for the favorable alleles (CC, 152 lines) contributed to a 1.01% decrease in ADF compared with the homozygous state of the unfavorable allele (TT, 24 lines). Out of two markers at QTL *QAdf/Adl.uga-2A*, one was linked to ADF (*S2A_711828719*) and one was linked to ADL (*S2A_711526601*), in both cases the lines carrying “G” allele (111 and 104 lines) had contributed to the decrement of ADF by 0.35% and ADL by 0.07%, indicating G to be consistently the favorable allele with lower ADF and ADL content at this locus (**Table 3**). At *QAdl.uga-5A* (*S5A_682890015*), “T” was considered the favorable allele with 24 lines, as it contributed to the decrement of ADL by 0.07% in comparison to the homozygous state of the unfavorable allele (CC, 157 lines). Two QTLs on chromosome 5B, *QAdl.uga-5B.1* (*S5B_605517698*) and *QAdl.uga-5B.2* (*S5B_486483108*) also showed a reduction in ADL by 0.05% in lines carrying “G” and “T” allele (19 and 89 lines, respectively) in comparison to the unfavorable allele (AA and CC). Interestingly, at *QAdl.uga-5B.3* (*S5B_607020013*), the “A” allele was considered a favorable homozygous allele (AA, 19 lines) and produced slightly lower ADL by 0.05% than the homozygous state of the unfavorable allele (GG, 153 lines). One of the strongest effects was observed at *QAdl.uga-6B* (*S6B_6242037*), where the lines with homozygous favorable allele (TT, 2 lines) had 0.2% of reduced ADL than the homozygous unfavorable allele (CC, 160 lines) (**Table 3**).

### 3.6 Additive effect of favorable alleles at major-effect QTLs

**Figure 3:**
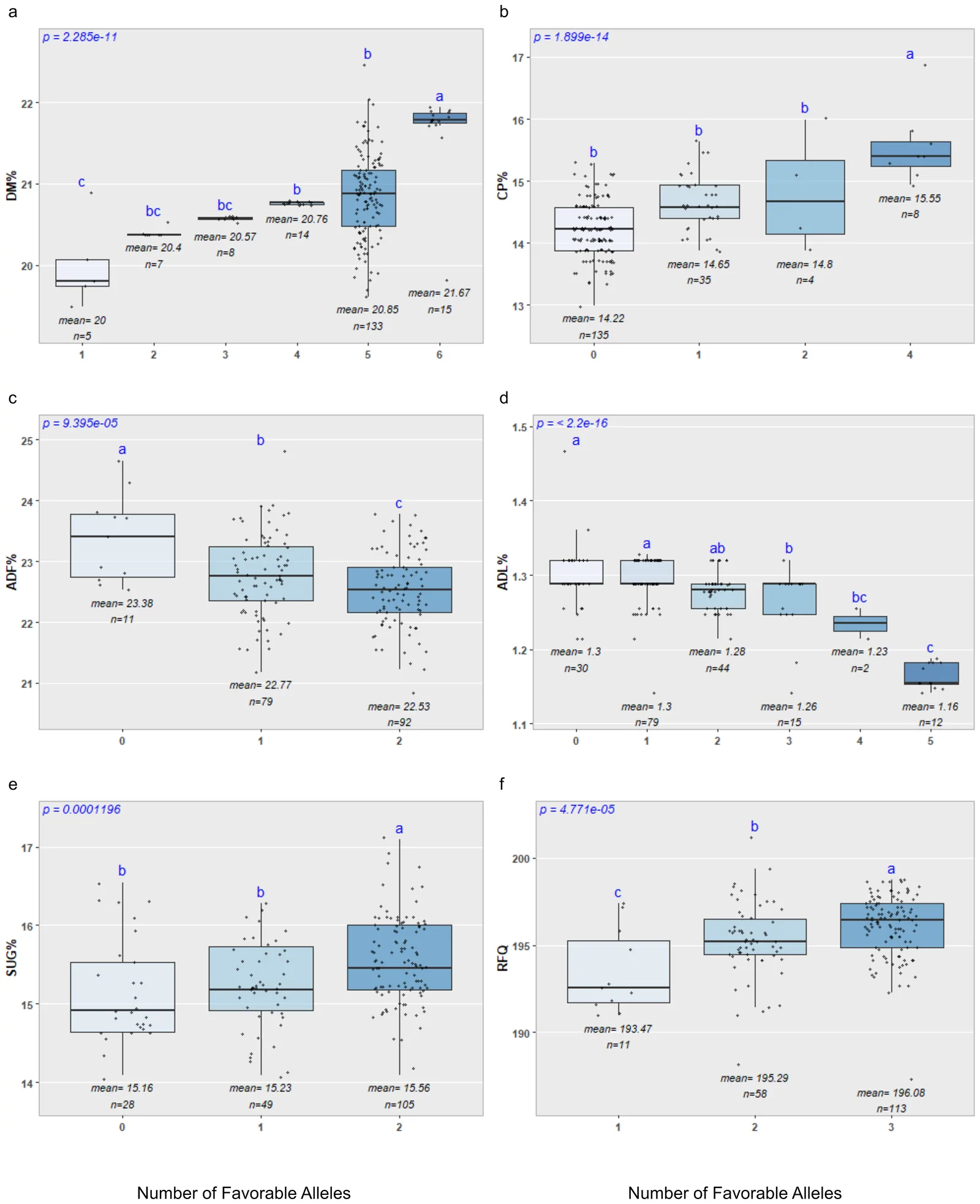
The relationship between the number of favorable alleles vs the BLUP values of, **(a)** DM, dry matter; **(b)** CP, crude protein; **(c)** ADF, acid detergent fiber; **(d)** ADL, acid detergent lignin; **(e)** SUG, sugar; **(f)** RFQ, relative forage quality. Boxes represent the distribution of BLUP values for wheat lines with the indicated numbers of favorable alleles. Individual points represent BLUP values of individual wheat lines. Mean values are shown below each box, and the associated letter above the box shows the TUKEY’s HSD results where different letters indicate a significant difference (p<0.05). The value ‘N’ below each box indicates the number of wheat lines within each favorable allele class.

Based on the cumulative number of favorable alleles, distinct genotype classes were identified for each trait, comprising six groups for DM, four for CP, three for ADF, six for ADL, and three groups each for SUG and RFQ (**Figure 3**). Across all traits, lines carrying the highest number of favorable alleles differed significantly (p < 0.05) from those with the lowest or null allele counts, demonstrating clear additive effects of allele pyramiding.

No lines were identified with zero favorable alleles for DM. However, genotypes harboring only a single favorable allele exhibited significantly lower DM content than lines pyramided with up to 6 favorable alleles (**Figure 3**). The cumulative stacking of favorable alleles across nine QTLs *QDm.uga-1A.1* through *QDm.uga-1A.7*, *QDm.uga-3A*, and *QDm.uga-3D* resulted in an overall increase of approximately 1.67% in DM. For RFQ, the highest values were observed in genotypes carrying three favorable alleles derived from three QTLs *QRfq.uga-1B*, *QRfq.uga-3B.1*, *QRfq.uga-3B.2*, corresponding to an increase of 2.61 RFQ units. Although no genotypes lacking favorable alleles were detected, lines possessing at least one favorable allele displayed comparatively lower RFQ values than those with the full favorable allele complement (**Figure 3**).

Similarly, genotypes carrying two favorable alleles exhibited significantly higher SUG content than those lacking them. By stacking favorable alleles from two QTLs *QSug.uga-4B* and *QDm/Sug.uga-7A* resulted in an approximate 0.4% increase in SUG. For ADF, the presence of a single favorable allele was sufficient to reduce fiber concentration by 0.61%, while stacking favorable alleles from QTLs *QAdf.uga-1B* and *QAdf/Adl.uga-2A* further enhanced this effect, resulting in a total reduction of 0.85%. In the case of ADL, genotypes carrying five favorable alleles across six QTLs *QAdf/Adl.uga-2A*, *QAdl.uga-5A*, *QAdl.uga-5B.1*, *QAdl.uga-5B.2*, *QAdl.uga-5B.3*, and *QAdl.uga-6B* exhibited a significant reduction of 0.14% in ADL compared with lines possessing fewer favorable alleles. Finally, stacking four favorable alleles across *QCp.uga-2D*, *QCp.uga-3A*, *QCp.uga-4A.1*, *QCp.uga-4A.2*, and *QCp.uga-4A.3* resulted in a cumulative increase of 1.33% in CP, confirming the positive impact of allele accumulation on forage protein concentration (**Figure 3**).

### 3.7 Candidate gene discovery

Candidate genes were identified for six of the 27 major-effect QTLs, *QRfq.uga-3B.1*, *QCp.uga-4A.1*, *QCp.uga-4A.3*, *QSug.uga-4B*, *QAdl.uga-5B.1*, and *QAdl.uga-5B.2* (**Table 2**). QTL *QRfq.uga-3B.1* for RFQ was associated with candidate gene *TraesCS3B02G356400*, which encodes a small regulatory protein involved in transcriptional control or nucleic acid binding. In contrast, for CP, *QCp.uga-4A.1* was associated with *TraesCS4A02G437000*, encoding a cytochrome P450 monooxygenase with iron-ion binding activity, a protein class known to participate in secondary metabolism and biosynthetic pathways influencing plant composition. Another CP-associated QTL, *QCp.uga-4A.3*, harbored *TraesCS4A02G019500*, which encodes a pentatricopeptide repeat (PPR) protein involved in translational regulation within mitochondria and/or chloroplasts (**Table 2**). QTL for SUG, *QSug.uga-4B* co-localized with *TraesCS4B02G396400*, annotated as a UDP-glucosyltransferase/glycogen phosphorylase-related protein. Lastly, ADL-linked QTL, *QAdl.uga-5B.1* was associated with *TraesCS5B02G430200*, encoding an A20/AN1-type zinc finger stress-associated protein (SAP), a regulatory protein implicated in growth regulation and stress signaling pathways. Additionally, *QAdl.uga-5B.2* co-localized with *TraesCS5B02G302000*, which encodes an RNA recognition motif (RRM) containing protein (**Table 2**).

## 4 Discussion

### 4.1 Phenotypic traits and their heritability

The GWAS study comprising 182 elite SRWW lines developed by various states in the southern U.S. was evaluated for eight forage-quality traits. All quality traits showed significant differences among the wheat genotypes for all the datasets used except DM and SUG for GAP-24, RFQ for GAP-25, and DM and ADL for IHF-25. However, due to large environmental variations, the JPC-25 location showed non-significant differences among genotypes for all forage-quality traits (**Supplementary Table 1**). Therefore, this location was dropped from the analysis. The lack of genotypic significance at JPC-25 indicates that environmental variance across location-year effects obscured the underlying genetic effects for forage-quality traits. Such environmental effects may arise from spatial and temporal variability in rainfall, temperature, soil properties, plant density, grazing or clipping regimes, and slight differences in developmental timing at harvest, all of which influence tissue moisture, nitrogen content, and cell-wall composition. Consequently, JPC-25 represents a pronounced G x E interaction, wherein environmental heterogeneity reduced the statistical power to discriminate genotypes for nutritive-value traits. These observations indicate that trait expression in this and similar datasets is strongly environment-dependent, emphasizing the need to interpret genetic effects within the context of environmental variation (Annicchiarico, 2002). The ADF and NDF are among the most important traits for determining forage quality, as they are negatively correlated with forage digestibility (Surber et al., 2011). According to Van Saun (2023), ADF and NDF below 35% and 50%, respectively are generally considered high-quality forage for all cool-season grasses. Results from this study showed mean values of ADF (18.47% to 26.59%), NDF (43.79% to 47.89%), and ADL (1.33% to 5.07%) across all datasets studied, with the lowest mean values observed at the GAP-25 location (**Supplementary Table 1**). Hence, our findings agree with previous studies (Van Saun, 2023; Kim and Anderson, 2015; Mullenix et al., 2014; and West et al., 2024), which observed similar results for these traits. West et al. (2024) mentioned that the wheat forage-quality traits values are comparable to those of annual ryegrass and other cool-season forages grazed in the SE U.S. In addition, wheat generally is higher in quality than annual ryegrass, triticale, and oat, and yields more DM per unit area than barley (Watson et al., 1993). Further, we observed DM and CP mean values ranging from 16.15% to 22.17% and 14.86% to 25.37%, respectively, which are higher than those reported by Kim and Anderson (2015) and West et al. (2024) for wheat. We also observed that the TDN mean ranged from 62.62% to 71.65%, with the higher TDN value observed at the GAP-25 location. Our TDN results were also in agreement with those reported by Kim and Anderson (2015), even greater, in some cases, than the mean TDN (66.9%) they reported. We also observed that SUG and RFQ ranged from 11.08% to 15.87% and from 126.17 to 196.28, respectively (**Supplementary Table 1**). These values could not be compared to previous findings due to lack of research on these traits. However, higher values of SUG and RFQ were directly associated with good-quality forage, as well established in previous studies (Miller et al., 2001; Undersander et al., 2002). Overall, these results indicate that the SRWW panel produced forage with consistently high nutritive value, with quality traits generally within or exceeding ranges reported for wheat and other winter annual forages harvested at vegetative to early reproductive stages (Kim and Anderson, 2015; Van Saun, 2023). This is particularly important for dual-purpose wheat systems in the SE U.S., where acceptable forage must provide both high intake potential and adequate digestibility before the crop shifts toward grain development (Mullenix et al., 2014; West et al., 2024; Van Saun, 2023). In this study the CP, ADF, NDF, ADL, TDN, and SUG traits showed overall moderate to high H^2^, whereas the traits, DM and RFQ showed low H^2^ (**Table 1**). These findings are consistent with the reports by Maulana et al. (2019) and Joshi et al. (2019), which show a comparable pattern of H^2^. Notably, ADF and NDF emerged as highly heritable traits (Maulana et al., 2019), demonstrating their value as reliable indicators of forage quality in breeding programs. The predominance of genetic control indicates that these traits can be effectively targeted in selection methods. Harnessing their H^2^ should facilitate genetic improvement of forage-quality in dual-purpose wheat breeding programs. The relatively low H^2^ observed for DM and RFQ is expected, as DM is highly influenced by environmental factors such as plant water status, harvest timing, and microclimatic variation, while RFQ integrates multiple intake and digestibility-related traits, making it sensitive to accumulated environmental and measurement effects (Undersander et al., 2002). As a result, direct phenotypic selection for these traits is less efficient, particularly in early-generation or minimally replicated trials, and genomic selection is preferred to capture the combined effects of many small-effect loci (Maulana et al., 2019). In contrast, ADF and NDF are structural cell-wall components with more stable expression across environments, leading to higher H^2^ (**Table 1**). Their strong genetic contribution and consistent performance make them reliable targets for direct selection and for calibrating genomic prediction models, with ADF closely linked to digestibility and NDF to voluntary intake (Hatfield et al., 2017). Consequently, ADF and NDF offer the clearest opportunities for repeatable selection and improvement of forage quality in dual-purpose wheat breeding (Meuwissen et al., 2001; Maulana et al., 2019).

### 4.2 Association among forage quality traits

The significant correlations among forage-quality traits we observed confirm many previous reports on wheat and other forage grasses. In our study, DM exhibited negative correlations with CP, ADF, and NDF, with r values ranging from -0.28 to -0.35 (**Figure 1**). These results were consistent with the findings of Maulana et al. (2019) and Colas et al. (2022), who observed a similar pattern, suggesting that increasing DM may be associated with reduced protein and fiber content. DM showed a significant positive association with SUG (r = 0.42), which contributed to superior forage quality. This result confirmed the previous findings by Kim and Anderson (2015). These trait relationships are consistent with changes in plant tissue composition as biomass shifts from young, leaf-rich, highly hydrated to more mature tissue with higher DM and altered carbon allocation. This highlights a breeding trade-off: improving DM alone is not always desirable if the gain is largely maturity-driven and is accompanied by declines in CP or less favorable fiber. Therefore, improvements in DM should be pursued in balance with CP, fiber fractions, and energy-related traits rather than as an isolated selection target.

As plants mature, fiber fractions tend to accumulate, accompanied by a corresponding reduction in CP. In this study, we observed significant negative association between CP and ADF (r = -0.40), NDF (r = -0.35), and ADL (r = -0.18). In contrast, CP showed a significant positive correlation with RFQ (r = 0.27), confirming previous results (Kim and Anderson, 2015; Capstaff and Miller, 2018), which reported that protein content declined as biomass accumulated, which tends to increase fiber content. Consequently, higher CP concentrations are associated with improved forage quality. The observed negative relationship between CP and fiber fractions reflects underlying plant developmental processes (Capstaff and Miller, 2018). During early vegetative growth, forage species contain more metabolically active tissue with higher protein, soluble nitrogen, and non-structural carbohydrates. As plants mature, carbon is increasingly partitioned to structural cell-wall components such as cellulose, hemicellulose, and lignin, while nitrogen concentration declines due to dilution and redistribution. In addition, the leaf-to-stem ratio generally decreases with maturity, with stems containing more structural carbohydrates and less protein than leaves (Ball et al., 2001). Thus, these changes explain the inverse relationship between CP and fiber, representing a shift from highly digestible, protein-rich tissue toward more structural and less digestible biomass (Van Soest et al., 1991; Capstaff and Miller, 2018).

The fiber components showed highly significant positive internal relations where ADF had a highly positive correlation with NDF (r = 0.95) and ADL (r = 0.36). As we expected, these fiber fractions showed significant negative correlation with TDN, SUG, and RFQ. Particularly, ADF correlation with TDN and SUG, r = - 0.60; NDF with TDN and SUG, r = - 0.69; NDF with RFQ r = - 0.50. Additionally, ADL showed significant negative correlations with TDN, SUG, and RFQ (r = -0.42, -0.35, and -0.36, respectively) (**Figure 1**). Similar results were also reported by Kim and Anderson (2015) and Hatfield et al. (2017) who showed lignin and fiber as primary constraints on digestibility and intake. These relationships have clear nutritional significance. NDF constitutes the bulk of the cell wall and largely determines rumen fill, thereby limiting voluntary intake, while ADF composed mainly of cellulose and lignin, is closely associated with digestibility (Saha et al., 2010). Lignin is indigestible and restricts microbial access to cell-wall carbohydrates, so increases in ADL disproportionately reduce TDN and RFQ. Consequently, higher structural fiber simultaneously lowers digestible energy and reduces animal intake, which explains why the fiber-rich tissues correspond to lower overall forage value (Van Soest et al., 1991; Hatfield et al., 2017).

For quality-improving traits, TDN and SUG were highly significant and positively correlated with RFQ (r = 0.87 and r = 0.57, respectively). As mentioned above, high SUG in grasses and small grain forages improves intake potential and overall feed value. Our results also underscore the importance of non-structural carbohydrates for increasing predicted energy and composite forage quality. This has also been observed by Miller et al. (2001); Wilkins and Lovatt (2011) and Capstaff and Miller (2018). In summary, the correlation network supports multi-trait selection strategies because structural fibers (NDF, ADF, and ADL) are strongly linked and collectively reduce RFQ and TDN (**Figure 1**). From a breeding perspective, the strong positive relationships of TDN and SUG with RFQ are promising, as they indicate that improvements in energy and overall forage value can be achieved simultaneously. Higher SUG enhances palatability and provides readily fermentable substrates for rumen microbes, while higher TDN reflects increased digestible energy supply. These correlations also suggest a practical route for indirect selection. Because RFQ is a composite index and showed lower H^2^ in this study, it may be more efficient to select indirectly through its component traits particularly lower ADF and NDF together with higher CP and SUG than to rely on RFQ alone. Such indirect selection aligns biological understanding with breeding efficiency by targeting the primary determinants of intake and digestibility rather than the composite index itself (Miller et al., 2001; Undersander et al., 2002; Capstaff and Miller, 2018).

### 4.3 Major-effect QTLs for forage quality traits

There are few studies using GWAS to identify and map MTAs, QTLs or SNPs linked to markers/genes controlling forage-quality traits in wheat or small grains in general. Our study discovered a total of 282 significant MTAs across 19 wheat chromosomes for forage-quality traits across all four datasets used in this research. PV explained by 282 MTAs ranged from 0.52% to 17.29%. These MTAs were resolved into 121 distinct QTLs; of these, 27 were considered major-effect QTLs, explaining ≥ 10% of PV (**Supplementary Table 2**).

We identified two major-effect QTLs that were associated with more than one trait. Among these, *QAdf/Adl.uga-2A* is associated with both ADF and ADL, and *QDm/Sug.uga-7A* linked with DM and SUG (**Table 2**). Similar multi-trait QTL associations have been recently reported in triticale (× *Triticosecale* Wittmack) by De Zutter et al. (2024). Similarly, a comparable association for ADF and ADL was reported for wheat straw fodder by Joshi et al. (2019). This is likely due to the fact that fiber and lignin content in forages are associated, as noted by Holtzapple (2003). These QTLs are more likely to reflect co-localized multi-trait associations arising from shared genetic control or tight linkage and represent practical targets for simultaneous improvement of correlated forage-quality traits in dual-purpose wheat breeding programs. In particular, *QDm/Sug.uga-7A* is notable, as sugar-related traits in wheat have previously been linked to chromosomal regions 7A, that regulate stem water-soluble carbohydrate accumulation and remobilization. While most prior work focused on reproductive-stage stem reserves rather than vegetative forage tissue (Dong et al., 2016; Li et al., 2020). Therefore, the co-localization of DM and SUG at this locus is biologically consistent, suggesting a potential role in carbohydrate partitioning, tissue maturation, or both.

We considered a QTL as stable when it was detected in at least two environments (datasets). Stable QTLs are valuable for marker-assisted selection (MAS) in breeding programs. These loci also provide a robust framework for dissecting plant adaptation to diverse environments. In this study, we found three stable major-effect QTLs, *QRfq.uga-3B.1, QRfq.uga-3B.2* and *QDm/Sug.uga-7A*. QTL *QDm/Sug.uga-7A* was particularly of interest since it was not only stable but also associated with multiple traits probably having a pleotropic effect and explaining 10.6% of PV for SUG, however, explaining only 6.4% PV for DM (**Table 2**). From a breeding standpoint, stability across environments substantially increases the value of these loci because environment-specific QTLs are less valuable to deploy in breeding programs. In our study, *QRfq.uga-3B.1, QRfq.uga-3B.2*, and *QDm/Sug.uga-7A* were detected consistently, so they should be prioritized for further validation and marker development (**Table 2**). The *QRfq.uga-3B* region is of particular interest because RFQ is composite index of intake and digestibility; therefore, any locus with repeated effects on RFQ across environments is potentially useful for improving forage value. However, GWAS alone cannot tell us if these multi-trait signals observed in this study represent true pleiotropy or tight linkage among neighboring genes. Resolving this requires follow-up genetic dissection. For instance, one could fine-map the locus in biparental or near-isogenic populations, perform haplotype-based association studies, examine gene expression in relevant tissues, and ultimately functional validation (Deng et al., 2011; Nyine et al., 2021).

The favorable alleles at 11 major-effect QTLs, present at high frequency across the panel, may indicate long-term directional selection pressure for these loci in SRWW breeding programs. High frequencies of advantageous alleles at major-effect QTLs have been reported in elite bread wheat germplasm (Pang et al., 2020), where they observed that favorable alleles for key agronomic traits were already common in elite breeding lines. This reflects a cumulative impact of long-term phenotypic selection, as these alleles moved toward fixation and an increase of their frequencies. The genetic variation surrounding these QTLs becomes increasingly reduced, which can hinder the detection of MTAs and often results in the exclusion of low-variability loci during their genotyping. The fixation of favorable alleles may also explain the high H^2^ estimates observed for several traits. Also, reduced allelic diversity at selected loci can lead to more consistent trait expression within breeding populations (Pang et al., 2020). In this diversity panel, the high frequency of favorable alleles likely reflects the fact that these are elite breeding lines which have been selected for adaptation and performance in the SE U.S. This is beneficial in the short term, as prior selection may have indirectly improved forage-quality traits. However, it also implies that future rates of gain within the same elite pool may diminish as these favorable alleles approach fixation. Accordingly, long-term improvement will likely require introgression of novel alleles from less-related germplasm, landraces, or pre-breeding material. Additionally, the relatively small panel size used here limits the power to detect rare or small-effect variants and may overestimate the effect sizes of significant loci. These considerations should guide the interpretation of major-effect QTL counts and the prioritization of loci for validation (Saini et al., 2022).

### 4.4 Additive effect of favorable alleles

Additive effect of favorable alleles evaluated cumulative effects of favorable alleles occurring naturally across the GWAS panel, meaning observed additive patterns may partly reflect breeding history, population structure, or linkage among favorable haplotypes, and rare or small-effect alleles may be underrepresented. Stratifying genotypes by cumulative number of favorable alleles revealed clear, progressive shifts in trait means, with genotypic classes with higher allele counts exhibiting statistically superior performance relative to those with fewer or no favorable alleles. Similar allele stacking strategies have been successfully implemented in wheat to improve grain quality attributes (Gautam et al., 2020; Gupta et al., 2022) as well as agronomic and yield-related traits (Wang et al., 2022; Subedi et al., 2024). In contrast, the application of QTL pyramiding for forage quality improvement in wheat has received comparatively limited attention. Thus, our GWAS study identified useful allelic associations among several forage quality traits for future use in breeding dual-purpose wheat.

DM responded positively to allele accumulation, with genotypes carrying stacked favorable alleles across nine QTLs on chromosomes 1A, 3A, and 3D (*QDm.uga-1A.1* through *QDm.uga-1A.7*, *QDm.uga-3A*, and *QDm.uga-3D*) exhibiting an overall increase of 1.67% in DM (**Figure 3**). Although the magnitude of change appears modest, incremental increases in DM are agronomically meaningful in winter annual forage systems, where moisture content directly influences harvest management, preservation efficiency, and voluntary intake. Comparable improvements through gene pyramiding have been reported for quality traits in rice, underscoring the broader applicability of this strategy across cereal crops (Gupta, 2007). In our study, genotypes harboring favorable alleles at three RFQ-associated QTLs (*QRfq.uga-1B*, *QRfq.uga-3B.1*, *QRfq.uga-3B.2*) recorded an average increase of 2.61 RFQ units compared with lines carrying fewer favorable alleles (**Figure 3**). Nevertheless, the superior performance of lines with the complete favorable allele set indicates that deliberate pyramiding can further increase forage quality beyond current levels. SUG increased by approximately 0.4% in genotypes combining favorable alleles at *QSug.uga-4B* and *QDm/Sug.uga-7A* compared with lines lacking these alleles. Higher soluble sugar is known to enhance palatability and energy supply in cool-season forages (Castillo, 2017). In contrast, ADF responded inversely, consistent with their established negative relationship with digestibility and intake. A single favorable allele at *QAdf.uga-1B* reduced ADF by about 0.61%, while stacking an additional favorable allele at *QAdf/Adl.uga-2A* further decreased ADF to a total of 0.85% (**Figure 3**). These reductions, though numerically small, are agronomically relevant. Extension guidelines frequently note that a 1-percentage-point decrease in ADF is associated with an appreciable increase in digestible dry matter and potential milk production in high-producing dairy cows (Newman et al., 2009).

The response of ADL further supports the functional relevance of the identified loci in modifying cell wall composition. Genotypes carrying five favorable alleles across six ADL-associated QTLs (*QAdf/Adl.uga-2A*, *QAdl.uga-5A*, *QAdl.uga-5B.1* to *QAdl.uga-5B.3*, *QAdl.uga-6B*) showed a 0.14% reduction in ADL compared with lines with fewer favorable alleles. Stacking favorable alleles at five CP-associated QTL (*QCp.uga-2D*, *QCp.uga-3A*, *QCp.uga-4A.1* to *QCp.uga-4A.3*) resulted in a cumulative increase of 1.33% (**Figure 3**). Given that cereal forages often approach the lower threshold of protein required for optimal rumen function, such gains can meaningfully reduce dependence on supplemental protein sources. Comparable additive effects of multiple loci on protein-related traits have been reported in wheat grain quality studies (Gautam et al., 2020). Collectively, these results align with a growing body of evidence demonstrating that pyramiding favorable alleles at major-effect QTL can yield cumulative, largely additive gains in complex agronomic traits (Zhang et al., 2013). From a breeding perspective, the observed trait shifts are modest but meaningful, as they moved all key forage-quality traits in a favorable direction. Increases of approximately 1.33% in CP, 1.67% in DM, reductions of 0.85% in ADF, and measurable improvements in RFQ and SUG could be valuable if consistently reproduced across breeding populations, particularly when achieved without compromising adaptation and grain production. In dual-purpose wheat systems, even incremental gains in digestibility and intake potential can improve livestock performance during the winter grazing period. The simultaneous improvement of DM, CP, ADF, ADL, SUG, and RFQ underscores the potential of MAS as a targeted breeding strategy for developing high-quality dual-purpose and forage-oriented wheat cultivars. The practical application of MAS for these traits depends on the number of loci involved, the magnitude and stability of their effects, and the feasibility of developing robust, breeder-friendly assays (Chukwu et al., 2019). In dual-purpose wheat breeding, MAS is likely most effective for the small set of stable, moderate-effect loci (such as QDm/Sug.uga-7A and QRfq.uga-3B.1 & 2) while the broader polygenic component of forage quality will be better addressed by genomic selection. Prior to large-scale implementation, the additive effects observed here should be validated in segregating populations and across multiple environments to confirm that favorable allele combinations remain stable and consistently beneficial beyond the discovery panel.

### 4.5 Gene candidacy and novelty test

One of the objectives of our study was to identify novel QTLs underlying wheat forage-quality traits. To this end, we compared the physical locations of previously reported loci in the wheat genome with the major-effect QTLs identified in this study. The novelty assessment was expended beyond forage-quality studies to also include recent wheat GWAS and QTL reports for grain quality, yield components, plant height, lodging resistance, and developmental traits. This comparison leveraged reference-genome-anchored resources from publicly available databases, including GrainGenes, Ensembl Plants, URGI/PlantBioinfoPF and NCBI, to ensure accurate cross-study alignment and reduce ambiguity from historical marker positions (Alaux et al., 2018, 2023). In particular, a previous study by Joshi et al. (2019) reported significant SNPs for ADF, NDF, and ADL on chromosomes 1A, 2B, and 3A. Likewise, in this study, we identified SNPs associated with ADF on chromosome 2B, however, they didn’t meet the threshold for major-effect QTLs. Comparative analysis further revealed partial co-localization with previously reported adaptive loci on chromosome 1A. Specifically, Zhang et al. (2024) identified a multi-effect QTL associated with lodging resistance and plant height on 1A region (296.9-297.7 Mb). The QTL *QDm.uga-1A.2* identified in this study for DM located at 297.74 Mb (S1A_297738248), co-localizes with this region. In addition, QTL and meta-QTL studies have frequently reported broad confidence intervals encompassing the distal region of chromosome arm 1AL. The DM-associated QTL *QDm.uga-1A.5*, mapped at 305.11 Mb (S1A_305110930), falls within the same QTL-rich region on the long arm of 1A that has been implicated in grain yield components and end-use quality (Naraghi et al., 2019; Miao et al., 2022). Numerous studies have investigated stem carbohydrate reserves, particularly water-soluble carbohydrates, including fructans, and have identified multiple MTAs/QTLs distributed across the wheat genome (Bellucci et al., 2015; Dong et al., 2016; Li et al., 2020; Guerra et al., 2021). The QTL on chromosome 1A for DM should be interpreted with caution because DM at the vegetative forage stage is an integrative phenotype influenced by biomass accumulation, tissue water status, and maturity-related changes in plant structure, the QDm.uga-1A.2 locus may reflect pleiotropic developmental effects or tight linkage with loci affecting plant height, lodging adaptation, or grain yield rather than a direct forage-quality-specific biochemical determinant. Thus, the 1A DM locus is best regarded as a putative forage-quality QTL that will require validation under joint phenotyping for developmental and compositional traits before a trait-specific interpretation can be made.

Despite these regional associations, no previously published MTAs/QTLs were found to directly overlap the major-effect QTLs identified for forage-quality traits in the present study. Consequently, the remaining 25 major-effect QTLs detected here are considered putative novel loci contributing to forage-quality variation in wheat. Here, the term “putative novel” is used in a conservative manner: it indicates the absence of direct physical overlap with previously reported loci based on expanded reference-genome-based comparison. This is not the stronger claim that these loci are entirely unrelated to all prior reported agronomic or developmental regions. This distinction is important because many published wheat QTLs span broad intervals and represent agronomic, yield, or grain-quality phenotypes rather than fresh-forage quality measured in dual-purpose wheat.The identification of candidate genes enables the refinement of broad QTL regions into a limited number of genes that to underlie control the traits of interest. Nevertheless, establishing biologically meaningful candidate genes remains challenging and requires careful integration of genetic, functional, and literature-based evidence. In the present study, candidate genes were successfully identified for six of the 27 major-effect QTLs detected, including loci associated with RFQ, CP, SUG, and ADL (**Table 2**). The identified genes predominantly encode regulatory or metabolic proteins, underscoring the importance of transcriptional, post-transcriptional, and biochemical control in governing complex forage quality traits. Moreover, the functional roles of some proteins encoded by these candidate genes have previously been linked to physiological processes relevant to quality determination in plants. For instance, *TraesCS5B02G430200* identified as a candidate gene for ADL-associated QTL *QAdl.uga-5B.1*, encodes an A20/AN1-type zinc finger stress-associated protein (**Table 2**). Members of this protein family have been reported to play critical roles in abiotic stress tolerance by regulating growth and stress-responsive signaling pathways (Singh, 2024). Accumulating evidence suggests that abiotic stress can indirectly influence cell wall composition and lignification by regulating stress-responsive developmental pathways, thereby affecting lignin content in plants (Denness et al., 2011; Choi et al., 2023). *TraesCS5B02G430200* is biologically plausible even if its effect on ADL is indirect. Similarly, *TraesCS4A02G019500*, the candidate gene associated with CP-related QTL *QCp.uga-4A.3*, encodes a pentatricopeptide repeat (PPR) protein (**Table 2**). PPR proteins are well known for their roles in RNA editing, splicing, stabilization, and translation within mitochondria and chloroplasts. Through effects on chloroplast and mitochondrial gene expression, this locus could alter photosynthetic efficiency, respiration, metabolic balance, and tissue development, thereby affecting CP indirectly through carbon–nitrogen relationships rather than by directly encoding a storage-protein factor (Barkan and Small, 2014: Qi et al., 2019) LD in wheat often extends beyond individual SNP intervals, genome annotations remain incomplete in some regions, and the nearest annotated gene may not be the causal locus. The present study evaluated an elite wheat lines rather than near-isogenic or biparental fine-mapping material, candidate-gene assignment based solely on physical proximity should be treated as a prioritization step rather than proof of causality. Their functions suggest potential effects on forage quality, either directly through carbohydrate metabolism, carbon partitioning, cell-wall formation, or lignification or indirectly, by influencing developmental timing, or stress responses that affect CP, SUG, or fiber accumulation. Future validation should integrate fine-mapping with transcriptomic, allele-specific expression studies, and functional assays to confirm causal roles (Deng et al., 2011; Nyine et al., 2021).

### 4.6 Importance for breeding for forage quality

This work adds to the limited number of studies that have utilized GWAS to dissect the genetic basis of forage-quality in wheat. Consistent with results observed for other small grains, most of the MTAs detected in our present study had small effects, with few major-effect QTLs contributing to individual traits. This reflects the genomic complexity and polygenic nature of forage-quality traits, governed by many genes with small effects, as confirmed in this study. Such genome complexity is also likely influenced by long-term selection in wheat breeding programs, where quality traits have been indirectly improved alongside agronomic traits. Similar findings were recently reported by De Zutter et al. (2024) and Gari et al. (2025) in triticale and oat, where the majority of minor effect QTLs were detected, demonstrating the genetic complexity governing the forage quality traits in small-grain crops.

This genetic architecture has clear implications for breeding strategies. Major, stable loci are valuable targets for MAS but do not account for sufficient genetic variance to achieve sustained gain on their own. Consequently, forage-quality improvement in dual-purpose wheat will likely benefit most from combining MAS for a limited set of validated stable loci with genomic selection to capture the numerous small-effect loci underlying the polygenic background. This is especially critical for traits such as DM and RFQ, where low H^2^ and high environmental sensitivity limit the efficiency of direct phenotypic selection.

Wheat has a complex, large genome (~17 Gb) and is allohexaploid, comprising three distinct subgenomes (A, B, and D), and an incompletely resolved genome assembly complicates marker discovery and QTL mapping of traits (Sukumaran and Yu, 2013). Our study demonstrated that most of the MTAs were located on B and D subgenomes, indicating that these subgenomes exhibit a higher level of polymorphism and contribute significantly to the genetic variation underlying forage-quality traits (**Supplementary Table 2**). Similar results were reported by Joshi et al. (2019), who detected MTAs for straw fodder quality traits on the B and A subgenome, further showing the prominent role of the B genome in governing forage-quality in wheat. However, the GWAS diversity panel size (182 lines) used in this study, may have contributed to the relatively limited number of major-effect QTLs identified, also highlighted by Saini et al. (2022) in their review, where they emphasized the influence of population size on GWAS power. Importantly, this study presents elite lines of SRWW carrying favorable alleles at the 11 major-effect QTLs, offering direct, useful materials for dual-purpose wheat development programs. The next step should be conversion of the most reliable SNPs into breeder-friendly assays, preferably Kompetitive Allele-Specific PCR (KASP) markers, for routine use in parental screening and early-generation selection. In hexaploid wheat this step requires very careful assay design, because homeologous genome sequences can otherwise cause cross‐amplification and false clustering. Therefore, marker development should be accompanied by independent validation across contrasting genetic backgrounds. For instance, Makhoul et al. (2020) showed that candidate KASP assays were tested in a panel of 213 diverse wheat lines from major growing regions worldwide, as well as in multiple backcross families, to confirm robustness. In our case, we suggest prioritizing KASP development for the *QRfq.uga-3B.1*, *QRfq.uga-3B.2*, and *QDm/Sug.uga-7A* loci, since these QTLs showed repeated effects across multiple datasets and thus appear most promising for the SE U.S. dual‐purpose wheat breeding pipeline. Importantly, however, even these “stable” loci must be further vetted: before routine deployment, these markers should be validated across a broader set of dual-purpose germplasm and target environments to verify that their effect sizes hold up and that there is lack of unfavorable trade-offs.

These loci can be efficiently exploited through molecular assays, including KASP for single-marker diagnostics and higher-throughput genotyping platforms (SNP arrays, GBS) which enable breeders to screen progeny for desirable alleles throughout the breeding cycle. This MAS approach can streamline breeding efforts by reducing the time and labor required for chemical analysis of wheat forage-quality traits in the future. Nevertheless, routine breeding will remain challenging if selection relies solely on a few markers, because forage quality traits are controlled by many loci, subject to substantial G x E interactions, and exhibit strong interdependence among fiber fractions, CP, and energy components. Therefore, an effective breeding strategy for dual-purpose wheat in the SE U.S. will likely integrate validated KASP markers for stable major-effect QTLs with genomic prediction models trained on multi-environment phenotypes. This combined approach allows breeders to fix favorable alleles at key loci while simultaneously capturing the polygenic variation that underlies overall forage value.

## 5 Conclusion

Our study combining phenotypic analysis with GWAS offers new insights into the genetic basis of forage quality in wheat. We can highlight, particularly, the following:

➢ Overall, the results show that forage-quality traits in SRWW are under measurable genetic control, but that their expression is strongly conditioned by environment and by the biological relationships among CP, SUG, and fiber fractions (ADF, NDF, ADL).
➢ The correlation direction observed here confirms that fiber-related traits remain central constraints on digestibility, intake potential, CP, and overall forage value, whereas energy-related traits such as TDN and SUG contribute positively to overall forage quality.
➢ Allelic effect and favorable allele accumulation analyses demonstrated largely additive contributions of favorable alleles across major-effect QTLs. Genotypes carrying higher numbers of favorable alleles consistently exhibited improved forage-quality profiles, including increased CP, SUG, and RFQ, along with reduced ADF and ADL content.
➢ From a breeding standpoint, the most valuable outcome of this study is the identification of stable genomic regions, particularly *QRfq.uga-3B.1*, *QRfq.uga-3B.2*, and *QDm/Sug.uga-7A* which provide tractable targets for molecular improvement of forage quality in dual-purpose wheat. These loci are more valuable than environment-specific QTLs because they offer higher potential for conversion into breeder-friendly markers, validation across diverse germplasm, and deployment under the variable environmental conditions typical of the SE U.S.
➢ Candidate genes identified in this study encode regulatory and metabolic proteins involved in transcriptional control, stress-responsive signaling pathways, plant development, and metabolic regulation, underscoring their functional relevance to forage quality traits. Their characterization provides valuable targets for future functional validation and advances our understanding of the molecular mechanisms underlying forage quality in wheat.
➢ The predominance of small-effect loci and the low H^2^ indicate that forage quality is highly polygenic and cannot be optimized through a few markers alone. Consequently, the most effective breeding strategy will be to combine MAS for validated, stable major-effect loci with genomic selection to capture the remaining polygenic variation. Such an integrated approach is well aligned with dual-purpose wheat improvement in the SE U.S., where cultivars must simultaneously maintain forage quality, agronomic adaptation, and grain yield across diverse environments.
➢ This study strengthens the importance of incorporating forage-quality traits into mainstream SRWW breeding and identifies biologically relevant genomic regions to support that goal. However, additional validation across diverse germplasm and location-year is still needed, but the present results establish a useful framework for developing improved dual-purpose wheat cultivars for the SE U.S.

## Data Availability

Data are available at: DOI: 10.5061/dryad.p5hqbzkzr; DOI: 10.5061/dryad.wwpzgmsvk.

## References

[B1] Alaux M. Dyer S. Sen T. Z. (2023). “ Wheat data integration and FAIRification: IWGSC, GrainGenes, Ensembl and other data repositories,” in The Wheat Genome ( Springer International Publishing, Cham), 13–25. doi: 10.1007/978-3-031-38294-9_2

[B2] Alaux M. Rogers J. Letellier T. Flores R. Alfama F. Pommier C. . (2018). Linking the International Wheat Genome Sequencing Consortium bread wheat reference genome sequence to wheat genetic and phenomic data. Genome Biol. 19, 111. doi: 10.1186/s13059-018-1491-4 30115101 PMC6097284

[B3] Alqudah A. M. Sallam A. Baenziger P. S. Börner A. (2020). GWAS: Fast-forwarding gene identification and characterization in temperate cereals: lessons from barley, a review. J. Adv. Res. 22, 119–135. doi: 10.1016/j.jare.2019.10.013 31956447 PMC6961222

[B4] Annicchiarico P. (2002). Genotype × Environment Interactions: Challenges and Opportunities for Plant Breeding and Cultivar Recommendations (Rome: FAO). Available online at: https://books.google.com/books?hl=en&lr=&id=so4UJSzWNbIC&oi=fnd&pg=PR7&dq=Annicchiarico,+P.+(2002).+Genotype+%C3%97+Environment+Interactions:+Challenges+and+Opportunities+for+Plant+Breeding+and+Cultivar+Recommendations.+Rome:+FAO.&ots=dD5ilBqaUa&sig=_R3EgD5S7xWTR-lCcnUpyax9tEQ (Accessed April 3, 2026).

[B5] Appels R. Eversole K. Stein N. Feuillet C. Keller B. IWGSC (2018). Shifting the limits in wheat research and breeding using a fully annotated reference genome. Science 361, eaar7191. doi: 10.1126/science.aar7191 30115783

[B6] Ball D. M. Collins M. Lacefield G. D. Martin N. P. Mertens D. A. Olson K. E. . (2001). “ Understanding forage quality,” in American Farm Bureau Federation Publication, vol. 1. (Park Ridge, IL: American Farm Bureau Federation Publication), 1–15. Available online at: https://www1.agric.gov.ab.ca/$department/deptdocs.nsf/ba3468a2a8681f69872569d60073fde1/afac7f43bc58261987257a3000687206/$FILE/foragequality.pdf (Accessed April 3, 2026).

[B7] Barkan A. Small I. (2014). Pentatricopeptide repeat proteins in plants. Annu. Rev. Plant Biol. 65, 415–442. doi: 10.1146/annurev-arplant-050213-040159 24471833

[B8] Bates D. M. (2010). Lme4: Mixed-Effects Modeling With R. Available online at: http://lme4.r-forge.r-project.org/lMMwR/lrgprt.pdf (Accessed April 3, 2026).

[B9] Bates D. Mächler M. Bolker B. Walker S. (2015). Fitting linear mixed-effects models using lme4. J. Stat. Software 67, 1–48. doi: 10.18637/jss.v067.i01

[B10] Baxter L. Singleton T. R. (2025). “ Planting and growing annual forages in Georgia,” in Bulletin 1584. University of Georgia Cooperative Extension. (Athens, GA: University of Georgia Cooperative Extension). Available online at: https://fieldreport.caes.uga.edu/publications/B1584/annual-forages-in-georgia/ (Accessed April 3, 2026).

[B11] Bellucci A. Torp A. M. Bruun S. Magid J. Andersen S. B. Rasmussen S. K. (2015). Association mapping in scandinavian winter wheat for yield, plant height, and traits important for second-generation bioethanol production. Front. Plant Sci. 6, 1046. doi: 10.3389/fpls.2015.01046 26635859 PMC4660856

[B12] Boden S. A. McIntosh R. A. Uauy C. Krattinger S. G. Dubcovsky J. Rogers W. J. . (2023). Updated guidelines for gene nomenclature in wheat. Theor. Appl. Genet. 136, 72. doi: 10.1007/s00122-023-04253-w 36952017 PMC10036449

[B13] Bradbury P. J. Zhang Z. Kroon D. E. Casstevens T. M. Ramdoss Y. Buckler E. S. (2007). TASSEL: software for association mapping of complex traits in diverse samples. Bioinformatics 23, 2633–2635. doi: 10.1093/bioinformatics/btm308 17586829

[B14] Breseghello F. Sorrells M. E. (2006). Association mapping of kernel size and milling quality in wheat (Triticum aestivum L.) cultivars. Genetics 172, 1165–1177. doi: 10.1534/genetics.105.044586 16079235 PMC1456215

[B15] Capstaff N. M. Miller A. J. (2018). Improving the yield and nutritional quality of forage crops. Front. Plant Sci. 9, 535. doi: 10.3389/fpls.2018.00535 29740468 PMC5928394

[B16] Castillo M. (2017). Forage quality: concepts and practices. Available online at: http://www.ipni.net/publication/bettercrops.nsf/0/018FAAAB47082AFF852581D0004DCC51/$FILE/BC-2017-4.pdf (Accessed April 3, 2026).

[B17] Choi S. J. Lee Z. Kim S. Jeong E. Shim J. S. (2023). Modulation of lignin biosynthesis for drought tolerance in plants. Front. Plant Sci. 14, 1116426. doi: 10.3389/fpls.2023.1116426 37152118 PMC10157170

[B18] Chukwu S. C. Rafii M. Y. Ramlee S. I. Ismail S. I. Oladosu Y. Okporie E. . (2019). Marker-assisted selection and gene pyramiding for resistance to bacterial leaf blight disease of rice (Oryza sativa L.). Biotechnol. Biotechnol. Equip. 33, 440–455. doi: 10.1080/13102818.2019.1584054 37339054

[B19] Colas V. Barre P. van Parijs F. Wolters L. Quitté Y. Ruttink T. . (2022). Seasonal differences in structural and genetic control of digestibility in perennial ryegrass. Front. Plant Sci. 12, 801145. doi: 10.3389/fpls.2021.801145 35058960 PMC8765707

[B20] Deng S. Wu X. Wu Y. Zhou R. Wang H. Jia J. . (2011). Characterization and precise mapping of a QTL increasing spike number with pleiotropic effects in wheat. Theor. Appl. Genet. 122, 281–289. doi: 10.1007/s00122-010-1443-1 20872211

[B21] Denness L. McKenna J. F. Segonzac C. Wormit A. Madhou P. Bennett M. . (2011). Cell wall damage-induced lignin biosynthesis is regulated by a reactive oxygen species-and jasmonic acid-dependent process in Arabidopsis. Plant Physiol. 156, 1364–1374. doi: 10.1104/pp.111.175737 21546454 PMC3135913

[B22] De Zutter A. Piro M. C. Maenhout S. Maurer H. P. De Boever J. Muylle H. . (2024). Genome-wide association study for *in vitro* digestibility and related traits in triticale forage. BMC Plant Biol. 24, 223. doi: 10.1186/s12870-024-04927-7 38539072 PMC10976741

[B23] Dong Y. Liu J. Zhang Y. Geng H. Rasheed A. Xiao Y. . (2016). Genome-wide association of stem water soluble carbohydrates in bread wheat. PloS One 11, e0164293. doi: 10.1371/journal.pone.0164293 27802269 PMC5089554

[B24] Edwards J. T. Horn G. W. (2010). First Hollow Stem: A Critical Growth Stage for Dual-Purpose Producers. Fact Sheet PSS 2147 (Stillwater, OK: Okla. State Univ. Coop. Ext. Serv). Available online at: http://osufacts.okstate.edu/docushare/dsweb/Get/Document-6693/PSS_2147web.pdf (Accessed April 3, 2026).

[B25] Esposito S. Taranto F. Vitale P. Ficco D. B. M. Colecchia S. A. Stevanato P. . (2022). Unlocking the molecular basis of wheat straw composition and morphological traits through multi-locus GWAS. BMC Plant Biol. 22, 519. doi: 10.1186/s12870-022-03900-6 36344939 PMC9641881

[B26] Gari A. T. Ashenafi L. Dejene M. Mohammed K. Mekonen T. Feyisa F. . (2025). Genome-wide association mapping for grain and forage quality traits in a subtropical oat germplasm collection adapted to highland regions of Eastern Africa. bioRxiv, 2025–2011. doi: 10.1101/2025.11.26.690820 38621210

[B27] Gautam T. Dhillon G. S. Saripalli G. Rakhi Singh V. P. Prasad P. . (2020). Marker-assisted pyramiding of genes/QTL for grain quality and rust resistance in wheat (Triticum aestivum L.). Mol. Breed. 40, 49. doi: 10.1007/s11032-020-01125-9 30311153

[B28] Guerra F. P. Yáñez A. Matus I. Del Pozo A. (2021). Genome-wide association of stem carbohydrate accumulation and remobilization during grain growth in bread wheat (Triticum aestivum L.) in mediterranean environments. Plants 10, 539. doi: 10.3390/plants10030539 33809230 PMC8001439

[B29] Guo J. Khan J. Pradhan S. Shahi D. Khan N. Avci M. . (2020). Multi-trait genomic prediction of yield-related traits in US soft wheat under variable water regimes. Genes 11, 1270. doi: 10.3390/genes11111270 33126620 PMC7716228

[B30] Gupta P. K. (2007). “ Pyramiding genes/QTL for crop improvement using marker-aided selection (MAS),” in Search for New Genes. Eds. Chopra V. L. Sharma R. P. Bhat S. R. Prasanna B. M. ( Academic Foundation, New Delhi), 145–171.

[B31] Gupta P. K. Balyan H. S. Chhuneja P. Jaiswal J. P. Tamhankar S. Mishra V. K. . (2022). Pyramiding of genes for grain protein content, grain quality, and rust resistance in eleven Indian bread wheat cultivars: A multi-institutional effort. Mol. Breed. 42, 21. doi: 10.1007/s11032-022-01277-w 37309458 PMC10248633

[B32] Hatfield R. D. Rancour D. M. Marita J. M. (2017). Grass cell walls: a story of cross-linking. Front. Plant Sci. 7. doi: 10.3389/fpls.2016.02056 28149301 PMC5241289

[B33] Hill W. G. Weir B. S. (1988). Variances and covariances of squared linkage disequilibria in finite populations. Theor. Popul Biol. 33, 54–78. doi: 10.1016/0040-5809(88)90004-4 3376052

[B34] Hinson P. O. Pinchak B. Adams C. B. Jones D. Rajan N. Kimura E. . (2023). Forage and cattle production during organic transition in dual-purpose wheat systems. Agron. J. 115, 873–886. doi: 10.1002/agj2.21284 41531421

[B35] Holtzapple M. T. (2003). “ HEMICELLULOSES,” in Encyclopedia of Food Sciences and Nutrition ( Elsevier, Amsterdam, The Netherlands), 3060–3071. doi: 10.1016/B0-12-227055-X/00589-7

[B36] Jensen J. W. Magid J. Hansen-Moller J. Andersen S. B. Bruun S. (2011). Genetic variation in degradability of wheat straw and potential for improvement through plant breeding. Biomass Bioenergy 35, 1114–1120. doi: 10.1016/j.biombioe.2010.11.036 38826717

[B37] Joshi A. K. Kumar U. Mishra V. K. Chand R. Chatrath R. Naik R. . (2019). Variations in straw fodder quality and grain–straw relationships in a mapping population of 287 diverse spring wheat lines. F Crop Res. 243, 107627. doi: 10.1016/j.fcr.2019.107627 31853164 PMC6894307

[B38] Kaňovská I. Biová J. Škrabišová M. (2024). New perspectives of post-GWAS analyses: From markers to causal genes for more precise crop breeding. Curr. Opin. Plant Biol. 82, 102658. doi: 10.1016/j.pbi.2024.102658 39549685

[B39] Kim K. S. Anderson J. D. (2015). Forage yield and nutritive value of winter wheat varieties in the southern Great Plains. Euphytica 202, 445–457. doi: 10.1007/s10681-014-1325-8 30311153

[B40] Levene H. (1960). Robust tests for equality of variances, in Contributions to Probability and Statistics: Essays in Honor of Harold Hotelling, eds Olkin I. Ghurye S. G. Hoeffding W. Madow W. G. Mann H. B. . (Stanford, CA: Stanford University Press) 278–292.

[B41] Li H. Durbin R. (2010). Fast and accurate long-read alignment with Burrows–Wheeler transform. Bioinformatics 26, 589–595. doi: 10.1093/bioinformatics/btp698 20080505 PMC2828108

[B42] Li M. Liu Y. Ma J. Zhang P. Wang C. Su J. . (2020). Genetic dissection of stem WSC accumulation and remobilization in wheat (Triticum aestivum L.) under terminal drought stress. BMC Genet. 21, 50. doi: 10.1186/s12863-020-00855-1 32349674 PMC7191701

[B43] Lindedam J. Andersen S. B. DeMartini J. Bruun S. Jorgensen H. Felby C. . (2012). Cultivar variation and selection potential relevant to the production of cellulosic ethanol from wheat straw. Biomass Bioenergy 37, 221–228. doi: 10.1016/j.biombioe.2011.12.009 38826717

[B44] Lu Y. Ding Y. Xu C. Chen S. Xia C. Zhang L. . (2025). Identification of quality-related genomic regions and candidate genes in silage maize by combining GWAS and meta-analysis. Plants 14, 2250. doi: 10.3390/plants14152250 40805599 PMC12348138

[B45] Ma S. Wang M. Wu J. Guo W. Chen Y. Li G. . (2021). WheatOmics: A platform combining multiple omics data to accelerate functional genomics studies in wheat. Mol. Plant 14, 1965–1968. doi: 10.1016/j.molp.2021.10.006 34715393

[B46] Mahmud K. Lee K. Hill N. S. Mergoum A. Missaoui A. (2021). Influence of tall fescue epichloë Endophytes on rhizosphere soil microbiome. Microorganisms 9, 1843. doi: 10.3390/microorganisms9091843 34576739 PMC8468716

[B47] Mailhot D. J. Arrington J. Dunn D. Ware G. Buntin D. Babar M. (2024). Georgia 2023-2024 winter grain and forage performance tests. Agricultural experiment stations (CAES) university of Georgia. Annual publication 100-16. Available online at: https://site.extension.uga.edu/forageteam/files/2024/09/winter-crops-2024.pdf (Accessed April 3, 2026).

[B48] Makhoul M. Rambla C. Voss-Fels K. P. Hickey L. T. Snowdon R. J. Obermeier C. (2020). Overcoming polyploidy pitfalls: a user guide for effective SNP conversion into KASP markers in wheat. Theor. Appl. Genet. 133, 2413–2430. doi: 10.1007/s00122-020-03608-x 32500260 PMC7360542

[B49] Maulana F. Anderson J. D. Butler T. J. Ma X. F. (2019). “ Improving dual-purpose winter wheat in the southern great plains of the United States,” in Recent Advances in Grain Crops Research ( Intech Open, London, UK), 1–16. doi: 10.5772/intechopen.86417

[B50] Meuwissen T. H. Hayes B. J. Goddard M. (2001). Prediction of total genetic value using genome-wide dense marker maps. Genetics 157, 1819–1829. doi: 10.1093/genetics/157.4.1819 11290733 PMC1461589

[B51] Miao Y. Jing F. Ma J. Liu Y. Zhang P. Chen T. . (2022). Major genomic regions for wheat grain weight as revealed by QTL linkage mapping and meta-analysis. Front. Plant Sci. 13, 802310. doi: 10.3389/fpls.2022.802310 35222467 PMC8866663

[B52] Miller L. A. Moorby J. M. Davies D. R. Humphreys M. O. Scollan N. D. MacRae J. C. . (2001). Increased concentration of water-soluble carbohydrate in perennial ryegrass (Lolium perenne L.): milk production from late-lactation dairy cows. Grass Forage Sci. 56, 383–394. doi: 10.1046/j.1365-2494.2001.00288.x 37945311

[B53] Mullenix M. K. Dillard S. L. Lin J. C. Gamble B. E. Muntifering R. B. (2014). Evaluation of wheat and triticale forage for stocker production in the Gulf Coast region. Prof. Anim. Sci. 30, 296–304. doi: 10.15232/S1080-7446(15)30120-0 42430884

[B54] Naraghi S. M. Simsek S. Kumar A. Al Rabbi S. H. Alamri M. S. Elias E. M. . (2019). Deciphering the genetics of major end-use quality traits in wheat. G3: Genes Genomes Genet. 9, 1405–1427. doi: 10.1534/g3.119.400050 30804024 PMC6505165

[B55] Newman Y. C. Adesogan A. T. Vendramini J. M. Sollenberger L. E. (2009). Defining forage quality. EDIS, 1–7. doi: 10.32473/edis-ag332-2009

[B56] Nguyen D. T. Gomez L. D. Harper A. Halpin C. Waugh R. Simister R. . (2020). Association mapping identifies quantitative trait loci for digestibility in rice straw. Biotechnol. Biofuels 13, 165. doi: 10.1186/s13068-020-01807-8 33062051 PMC7545568

[B57] Nyine M. Adhikari E. Clinesmith M. Aiken R. Betzen B. Wang W. . (2021). The haplotype-based analysis of Aegilops tauschii introgression into hard red winter wheat and its impact on productivity traits. Front. Plant Sci. 12, 716955. doi: 10.3389/fpls.2021.716955 34484280 PMC8416154

[B58] Pang Y. Liu C. Wang D. Amand P. S. Bernardo A. Li W. . (2020). High-resolution genome-wide association study identifies genomic regions and candidate genes for important agronomic traits in wheat. Mol. Plant 13, 1311–1327. doi: 10.1016/j.molp.2020.07.008 32702458

[B59] Piepho H. P. Möhring J. Melchinger A. E. Büchse A. (2008). BLUP for phenotypic selection in plant breeding and variety testing. Euphytica 161, 209–228. doi: 10.1007/s10681-007-9449-8 30311153

[B60] Qi J. Ni F. Wang X. Sun M. Cui Y. Wu J. . (2019). The anther-specific CYP704B is potentially responsible for MSG26 male sterility in barley. Theor. Appl. Genet. 132, 2413–2423. doi: 10.1007/s00122-019-03363-8 31209536

[B61] Saha U. K. Sonon L. S. Hancock D. W. Hill N. S. Stewart L. Heusner G. L. (2010). Common terms used in animal feeding and nutrition. Available online at: https://openscholar.uga.edu/record/23738?v=pdf (Accessed April 3, 2026).

[B62] Saini D. K. Chopra Y. Singh J. Sandhu K. S. Kumar A. Bazzer S. . (2022). Comprehensive evaluation of mapping complex traits in wheat using genome-wide association studies. Mol. Breed. 42, 1. doi: 10.1007/s11032-021-01272-7 37309486 PMC10248672

[B63] Severino da Silva L. Coleman A. García C. C. V. Giri S. (2026). Agronomic responses of wheat and oat cultivars under dual-purpose and grain production management strategies. Crops 6, 27. doi: 10.3390/crops6020027 30654563

[B64] Siahsar B. A. Peighambari S. A. Taleii A. R. Naghavi M. R. Nabipour A. Sarrafi A. (2009). QTL analysis of forage quality traits in barley (Hordeum vulgare L.). Cereal Res. Commun. 37, 479–488. doi: 10.1556/CRC.37.2009.4.1

[B65] Singh K. (2024). “ Understanding the genetic and physiological mechanisms of day and night-time heat stress tolerance related to photosynthesis and key agronomic traits in wheat (Triticum aestivum L.),” ( Washington State University). Available online at: https://www.proquest.com/docview/3071791421?fromopenview=true&pq-origsite=gscholar&sourcetype=Dissertations%20&%20Theses (Accessed April 3, 2026).

[B66] Subedi M. Bagwell J. W. Ghimire B. Lopez B. Sapkota S. Babar M. A. . (2024). Identifying genomic regions associated with key agro‐morphological traits in soft red winter wheat using genome‐wide association study. Crop Sci. 64, 2316–2335. doi: 10.1002/csc2.21261 41531421

[B67] Subedi M. Ghimire B. Bagwell J. W. Buck J. W. Mergoum M. (2023). Wheat end-use quality: State of art, genetics, genomics-assisted improvement, future challenges, and opportunities. Front. Genet. 13, 1032601. doi: 10.3389/fgene.2022.1032601 36685944 PMC9849398

[B68] Sukumaran S. Yu J. (2013). “ Association mapping of genetic resources: achievements and future perspectives,” in Genomics of Plant Genetic Resources: Volume 1. Managing, Sequencing and Mining Genetic Resources ( Springer Netherlands, Dordrecht), 207–235. doi: 10.1007/978-94-007-7572-5_9

[B69] Sulc R. M. Franzluebbers A. J. (2014). Exploring integrated crop–livestock systems in different ecoregions of the United States. Eur. J. Agron. 57, 21–30. doi: 10.1016/j.eja.2013.10.007 38826717

[B70] Surber L. Abdel-Haleem H. Martin J. Hensleigh P. Cash D. Bowman J. . (2011). Mapping quantitative trait loci controlling variation in forage quality traits in barley. Mol. Breed. 28, 189–200. doi: 10.1007/s11032-010-9473-6 30311153

[B71] Talbot M. (1997). “ Resource allocation for selection systems,” in Statistical Methods for Plant Variety Evaluation ( Springer Netherlands, Dordrecht), 162–174. doi: 10.1007/978-94-009-1503-9_10

[B72] Undersander D. Moore J. E. Schneider N. (2002). “ Relative forage quality,” in Focus Forag, vol. 4. (Madison, WI: University of Wisconsin Extension), 1–2. Available online at: https://bpb-us-e1.wpmucdn.com/blogs-dev.cornell.edu/dist/e/1628/files/2016/03/Relative-Forage-Quality-25jb11q.pdf (Accessed April 3, 2026).

[B73] USDA Economic Research Service (2026). “ Wheat Sector at a Glance,” ( United States Department of Agriculture). Available online at: https://www.ers.usda.gov/topics/crops/wheat/wheat-sector-at-a-glance (Accessed April 3, 2026).

[B74] USDA National Agricultural Statistics Service (2025a). “ 2025 state agriculture overview: alabama,” ( United States Department of Agriculture). Available online at: https://www.nass.usda.gov/Quick_Stats/Ag_Overview/stateOverview.php?state=ALABAMA (Accessed April 3, 2026).

[B75] USDA National Agricultural Statistics Service (2025b). “ 2025 state agriculture overview: Georgia,” ( United States Department of Agriculture). Available online at: https://www.nass.usda.gov/Quick_Stats/Ag_Overview/stateOverview.php?state=GEORGIA (Accessed April 3, 2026).

[B76] USDA National Agricultural Statistics Service (2025c). “ 2025 state agriculture overview: north carolina,” ( United States Department of Agriculture). Available online at: https://www.nass.usda.gov/Quick_Stats/Ag_Overview/stateOverview.php?state=North+Carolina&year=2025 (Accessed April 3, 2026).

[B77] USDA National Agricultural Statistics Service (2025d). “ 2025 state agriculture overview: South Carolina,” ( United States Department of Agriculture). Available online at: https://www.nass.usda.gov/Quick_Stats/Ag_Overview/stateOverview.php?state=SOUTH+CAROLINA (Accessed April 3, 2026).

[B78] USDA National Agricultural Statistics Service (2026). “ Crop production,” ( United States Department of Agriculture). Available online at: https://release.nass.usda.gov/reports/crop0526.pdf?utm (Accessed April 3, 2026).

[B79] Van Saun R. J. (2023). Trace mineral metabolism: the maternal-fetal bond. Veterinary Clinics: Food. Anim. Pract. 39, 399–412. doi: 10.1016/j.cvfa.2023.06.003 37442677

[B80] Van Soest P. V. Robertson J. B. Lewis B. A. (1991). Methods for dietary fiber, neutral detergent fiber, and nonstarch polysaccharides in relation to animal nutrition. J. Dairy Sci. 74, 3583–3597. doi: 10.3168/jds.S0022-0302(91)78551-2 1660498

[B81] Wang Z. Dhakal S. Cerit M. Wang S. Rauf Y. Yu S. . (2022). QTL mapping of yield components and kernel traits in wheat cultivars TAM 112 and Duster. Front. Plant Sci. 13, 1057701. doi: 10.3389/fpls.2022.1057701 36570880 PMC9768232

[B82] Wang H. Li K. Hu X. Liu Z. Wu Y. Huang C. (2016). Genome-wide association analysis of forage quality in maize mature stalk. BMC Plant Biol. 16, 227. doi: 10.1186/s12870-016-0919-9 27769176 PMC5073832

[B83] Wang J. Zhang Z. (2021). GAPIT version 3: boosting power and accuracy for genomic association and prediction. Genomics Proteomics Bioinf. 19, 629–640. doi: 10.1016/j.gpb.2021.08.005 34492338 PMC9121400

[B84] Watson S. L. Fjell D. L. Shroyer J. P. Bolsen K. Duncan S. (1993). “ Small Grain Cereals for Forage. Publication MF-1072,” (Mahattan, KS: Kanas State University Agricultural Experiment Station and Cooperative Extension Service). Available online at: https://krex.k-state.edu/server/api/core/bitstreams/8a3ddd44-748a-4c32-a42d-de2caf12d22f/content (Accessed April 3, 2026).

[B85] West M. H. Smith W. B. Mullenix M. K. Rabinowitz A. N. Dillard S. L. (2024). Evaluation of dual-purpose wheat varieties in the Southeast United States. Appl. Anim. Sci. 40, 446–455. doi: 10.15232/aas.2023-02450

[B86] Wilkins P. W. Lovatt J. A. (2011). Gains in dry matter yield and herbage quality from breeding perennial ryegrass. Ir. J. Agric. Food Res., 23–30. Available online at: https://www.jstor.org/stable/41348153 (Accessed April 3, 2026). 42430723

[B87] Xu S. (2003). Theoretical basis of the Beavis effect. Genetics 165, 2259–2268. doi: 10.1093/genetics/165.4.2259 14704201 PMC1462909

[B88] Zadoks J. C. Chang T. T. Konzak C. F. (1974). A decimal code for the growth stages of cereals. Weed Res. 14, 415–421. doi: 10.1111/j.1365-3180.1974.tb01084.x 40046247

[B89] Zhang H. Li Y. Liu W. Sun Y. Tang J. Che J. . (2024). Genetic analysis of adaptive traits in spring wheat in Northeast China. Life. 14, 168. doi: 10.3390/life14020168 38398677 PMC10890535

[B90] Zhang B. Shi W. Li W. Chang X. Jing R. (2013). Efficacy of pyramiding elite alleles for dynamic development of plant height in common wheat. Mol. Breed. 32, 327–338. doi: 10.1007/s11032-013-9873-5 23976874 PMC3748324

