# Supplementary material for: Dissecting the genetics of forage quality traits in soft red winter wheat in the U.S. southeast region
Source: Front Plant Sci. 2026 Aug 26;17:1893113. doi: 10.3389/fpls.2026.1893113 (PMC13553917; doi:10.3389/fpls.2026.1893113)
Supplement: Supplementary file 1 [file DataSheet1.docx]

**Supplementary Table 1:**

Descriptive statistics and analysis of variance for forage-quality traits studied on 182 soft red winter wheat lines.

| Trait-Environment | General statistics of population | | | | | ANOVA (p-value) | | | |
| --- | --- | --- | --- | --- | --- | --- | --- | --- | --- |
|  | mean | SD | CV% | min | max | Genotype (G) | GXE | EXR | Heritability |
| DM_IHF -25 | 22.17 | 2.13 | 9.62 | 13.70 | 34.67 | 0.57658 | x | x | 0.07965788 |
| DM_JPC-25 | 16.19 | 1.34 | 8.28 | 11.35 | 27.37 | 0.7477 | x | x | 0.04669274 |
| DM_GAP-25 | 20.79 | 1.69 | 7.69 | 12.15 | 29.37 | 0.0004851 *** | x | x | 0.4059216 |
| DM_GAP -24 | 17.80 | 2.07 | 11.65 | 13.00 | 34.53 | 0.3218 | x | x | 0.114302449 |
| DM_All-Combined | 19.24 | 1.82 | 9.45 | 11.35 | 34.67 | 0.99109 | 0.01302 * | < 2e-16 *** | 0.00052 |
| CP_IHF -25 | 14.86 | 1.75 | 11.79 | 10.40 | 28.45 | 0.01183 * | x | x | 0.3129166 |
| CP_JPC-25 | 25.37 | 2.62 | 10.35 | 18.00 | 32.24 | 1 | x | x | 0 |
| CP_GAP-25 | 18.89 | 2.43 | 12.85 | 10.58 | 27.63 | 0.0034 ** | x | x | 0.3541241 |
| CP_GAP -24 | 19.19 | 2.62 | 13.68 | 11.66 | 28.63 | 0.003871 ** | x | x | 0.35017252 |
| CP_All-Combined | 19.58 | 2.43 | 12.39 | 10.40 | 32.24 | 0.02375 * | 0.02617 * | < 2e-16 *** | 0.08563 |
| ADF_IHF -25 | 19.37 | 1.22 | 6.28 | 13.21 | 25.23 | 1.429e-13 *** | x | x | 0.6759899 |
| ADF_JPC-25 | 23.63 | 2.69 | 10.99 | 18.08 | 32.17 | 1 | x | x | 0 |
| ADF_GAP-25 | 18.47 | 1.04 | 5.62 | 13.34 | 24.18 | <2e-16 *** | x | x | 0.751434 |
| ADF_GAP -24 | 26.59 | 2.18 | 8.24 | 15.66 | 33.38 | 0.0205 * | x | x | 0.29163389 |
| ADF_All-Combined | 21.99 | 1.89 | 8.60 | 13.21 | 33.38 | 2.149e-12 *** | 0.03634 * | < 2.2e-16 *** | 0.24306 |
| NDF_IHF -25 | 45.14 | 1.75 | 3.88 | 36.41 | 54.79 | 1.771e-11 *** | x | x | 0.6388819 |
| NDF_JPC-25 | 49.53 | 3.79 | 7.47 | 40.97 | 62.88 | 1 | x | x | 0 |
| NDF_GAP-25 | 43.79 | 1.46 | 3.34 | 35.43 | 50.96 | <2e-16 *** | x | x | 0.7552834 |
| NDF_GAP -24 | 47.89 | 2.78 | 5.59 | 36.08 | 56.24 | 0.0417 * | x | x | 0.261642257 |
| NDF_All-Combined | 46.56 | 2.60 | 5.59 | 35.43 | 62.88 | 1.78e-07 *** | 0.01821 * | < 2.2e-16 *** | 0.325 |
| ADL_IHF -25 | 2.10 | 0.23 | 11.11 | 1.37 | 2.82 | 0.2016 | x | x | 0.1730182 |
| ADL_JPC-25 | 1.68 | 0.28 | 16.75 | 1.11 | 2.79 | 1 | x | x | 2.156762E-18 |
| ADL_GAP-25 | 1.33 | 1.29 | 14.78 | 0.68 | 2.37 | 3.906e-12 *** | x | x | 0.6517388 |
| ADL_GAP -24 | 5.07 | 0.41 | 8.05 | 3.90 | 6.88 | 0.02549 * | x | x | 0.28311546 |
| ADL_All-Combined | 2.54 | 0.29 | 11.57 | 0.68 | 6.88 | 0.5283025 | 0.0001251 *** | < 2.2e-16 *** | 0.00352 |
| TDN_IHF -25 | 68.19 | 1.25 | 1.83 | 62.59 | 71.7 | 1.307e-07 *** | x | x | 0.5482791 |
| TDN_JPC-25 | 67.79 | 1.06 | 1.56 | 63.84 | 70.36 | 0.4184 | x | x | 0.1132338 |
| TDN_GAP-25 | 71.65 | 0.76 | 1.06 | 68.49 | 74.68 | 1.297e-10 *** | x | x | 0.6216639 |
| TDN_GAP -24 | 62.62 | 2.04 | 3.26 | 57.33 | 69.12 | 0.03538 * | x | x | 0.26906868 |
| TDN_All-Combined | 67.54 | 1.36 | 2.02 | 57.33 | 74.68 | 0.3203 | 2.527e-08 *** | < 2.2e-16 *** | 0.02422 |
| SUG_IHF -25 | 14.05 | 1.08 | 7.72 | 10.16 | 17.79 | 2.138e-08 *** | x | x | 0.5702863 |
| SUG_JPC-25 | 11.35 | 0.84 | 7.43 | 9.04 | 13.99 | 0.4182 | x | x | 0.1134075 |
| SUG_GAP-25 | 15.87 | 0.97 | 6.11 | 12.11 | 19.31 | 8.69e-09 *** | x | x | 0.5797835 |
| SUG_GAP -24 | 11.08 | 0.96 | 8.71 | 8.42 | 14.11 | 0.08307. | x | x | 0.22740476 |
| SUG_All-Combined | 13.09 | 0.97 | 7.41 | 8.42 | 19.31 | 4.933e-07 *** | 4.298e-08 *** | < 2.2e-16 *** | 0.162 |
| RFQ_IHF -25 | 176.01 | 8.45 | 4.79 | 125.28 | 199.11 | 0.0006620 *** | x | x | 0.3988243 |
| RFQ_JPC-25 | 126.17 | 26.62 | 21.19 | 47.01 | 183.63 | 1 | x | x | 0 |
| RFQ_GAP-25 | 196.28 | 10.48 | 5.34 | 143.72 | 219.89 | 0.06049. | x | x | 0.2435803 |
| RFQ_GAP -24 | 139.42 | 18.43 | 13.22 | 61.94 | 187.98 | 0.005732 ** | x | x | 0.33777162 |
| RFQ_All-Combined | 159.47 | 17.69 | 11.09 | 47.01 | 219.89 | 0.90189 | 0.05929. | < 2e-16 *** | 0.00433 |

**Supplementary Table 2:**

Marker-trait associations (MTAs) identified for forage-quality traits. These MTAs were resolved into 121 QTLs. The major-effect QTLs with phenotypic variance (PV) explained >10% are highlighted in bold.

| **QTL name** | **SNP** | **Trait** | **Environment** | **MAF** | **P.value** | **Effect** | **PV** |  |
| --- | --- | --- | --- | --- | --- | --- | --- | --- |
| *QDm.uga-1A* | S1A_1846793 | DM | BLUP_GAP-24 | 0.30 | 3.4371E-05 | -0.04 | 3.98 |  |
| *QDm.uga-1A.1* | S1A_303679238 | DM | BLUP_GAP-25 | 0.058 | 2.52786E-05 | 0.43 | **11.02** |  |
| *QDm.uga-1A.2* | S1A_298116765 | DM | BLUP_GAP-25 | 0.061 | 3.86547E-05 | 0.41 | 5.10 |  |
|  | S1A_297381169 | DM | BLUP_GAP-25 | 0.052 | 4.52376E-05 | 0.43 | **10.81** |  |
|  | S1A_297738248 | DM | BLUP_GAP-25 | 0.064 | 8.2428E-05 | 0.39 | **10.99** |  |
| *QDm.uga-1A.3* | S1A_299486443 | DM | BLUP_GAP-25 | 0.061 | 3.86547E-05 | 0.41 | **12.09** |  |
|  | S1A_299105750 | DM | BLUP_GAP-25 | 0.064 | 9.36599E-05 | 0.38 | **11.15** |  |
| *QDm.uga-1A.4* | S1A_302922335 | DM | BLUP_GAP-25 | 0.061 | 3.86547E-05 | 0.41 | **10.99** |  |
|  | S1A_302922343 | DM | BLUP_GAP-25 | 0.061 | 3.86547E-05 | 0.41 | **10.99** |  |
| *QDm.uga-1A.5* | S1A_305110930 | DM | BLUP_GAP-25 | 0.061 | 3.86547E-05 | 0.41 | **11.02** |  |
| *QDm.uga-1A.6* | S1A_180001065 | DM | BLUP_GAP-25 | 0.052 | 4.52376E-05 | 0.43 | **11.55** |  |
| *QDm.uga-1A.7* | S1A_162319212 | DM | BLUP_IHF-25 | 0.081 | 2.7347E-05 | -0.11 | **12.36** |  |
| *QSug.uga-1A* | S1A_572337796 | SUG | BLUP_All | 0.308 | 6.951E-06 | 0.12 | 6.86 |  |
|  | S1A_572337800 | SUG | BLUP_All | 0.308 | 6.951E-06 | 0.12 | 6.86 |  |
|  | S1A_572389565 | SUG | BLUP_All | 0.351 | 5.739E-05 | 0.10 | 6.61 |  |
|  | S1A_572389598 | SUG | BLUP_All | 0.351 | 5.739E-05 | 0.10 | 6.61 |  |
|  | S1A_572495730 | SUG | BLUP_All | 0.331 | 2.165E-05 | 0.11 | 6.49 |  |
|  | S1A_572495802 | SUG | BLUP_All | 0.331 | 2.165E-05 | 0.11 | 6.49 |  |
|  | S1A_572501203 | SUG | BLUP_All | 0.331 | 3.773E-05 | 0.11 | 5.78 |  |
|  | S1A_572501248 | SUG | BLUP_All | 0.331 | 3.773E-05 | 0.11 | 5.78 |  |
|  | S1A_572501259 | SUG | BLUP_All | 0.331 | 3.773E-05 | 0.11 | 5.78 |  |
|  | S1A_572501262 | SUG | BLUP_All | 0.331 | 3.773E-05 | 0.11 | 5.78 |  |
|  | S1A_572679727 | SUG | BLUP_All | 0.331 | 2.596E-05 | 0.11 | 7.59 |  |
|  | S1A_572861364 | SUG | BLUP_All | 0.328 | 1.414E-05 | 0.11 | 7.44 |  |
|  | S1A_572495730 | ADL | BLUP_All | 0.331 | 9.0314E-05 | 0.03 | 1.04 |  |
|  | S1A_572495802 | ADL | BLUP_All | 0.331 | 9.0314E-05 | 0.03 | 1.04 |  |
|  | S1A_572495730 | TDN | BLUP_All | 0.331 | 9.0314E-05 | 0.03 | 3.38 |  |
|  | S1A_572495802 | TDN | BLUP_All | 0.331 | 9.0314E-05 | 0.03 | 3.38 |  |
| *QRfq.uga-1B* | S1B_151287916 | RFQ | BLUP_GAP-24 | 0.090 | 3.0266E-05 | 3.45 | **11.55** |  |
|  | S1B_151287920 | RFQ | BLUP_GAP-24 | 0.090 | 3.0266E-05 | 3.45 | **11.55** |  |
| *QNdf.uga-1B* | S1B_275578301 | NDF | BLUP_GAP-24 | 0.029 | 2.2378E-05 | 0.67 | **9.26** |  |
| *QAdf.uga-1B* | S1B_126990314 | ADF | BLUP_IHF-25 | 0.157 | 2.1447E-05 | -0.55 | **10.11** |  |
| *QDm.uga-2A* | S2A_666833982 | DM | BLUP_GAP-24 | 0.145 | 1.1475E-07 | 0.09 | 6.01 |  |
| *QDm.uga-2A* | S2A_711050754 | DM | BLUP_GAP-24 | 0.116 | 5.2336E-06 | -0.07 | 0.62 |  |
| *QAdf/Adl.uga-2A* | S2A_711828719 | ADF | BLUP_GAP-24 | 0.346 | 1.5539E-05 | -0.19 | **10.31** |  |
|  | S2A_711467491 | ADL | BLUP_GAP-24 | 0.381 | 3.52481E-05 | -0.03 | **10.08** |  |
|  | S2A_711526601 | ADL | BLUP_GAP-24 | 0.381 | 2.39817E-05 | -0.03 | **11.30** |  |
|  | S2A_711828719 | ADL | BLUP_GAP-24 | 0.346 | 5.95654E-05 | -0.03 | **10.60** |  |
| *QSug/Tdn.uga-2A* | S2A_715332273 | SUG | BLUP_IHF-25 | 0.227 | 3.6538E-05 | -0.33 | 4.62 |  |
|  | S2A_715332308 | SUG | BLUP_IHF-25 | 0.227 | 3.6538E-05 | -0.33 | 4.62 |  |
|  | S2A_715332273 | TDN | BLUP_IHF-25 | 0.227 | 5.3587E-05 | -0.34 | 2.64 |  |
|  | S2A_715332308 | TDN | BLUP_IHF-25 | 0.227 | 5.3587E-05 | -0.34 | 2.64 |  |
| *QSug/Ndf.uga-2A* | S2A_707204761 | SUG | BLUP_IHF-25 | 0.131 | 4.9876E-05 | -0.49 | 1.65 |  |
|  |  | NDF |  | 0.131 | 8.3786E-05 | 0.86 | 0.86 |  |
| *QDm.uga-2B.1* | S2B_28659848 | DM | BLUP_GAP-24 | 0.308 | 4.9025E-08 | -0.05 | 8.16 |  |
| *QSug.uga-2B.1* | S2B_51883471 | SUG | BLUP_GAP-24 | 0.343 | 9.8908E-05 | 0.08 | 3.76 |  |
|  | S2B_51883495 | SUG | BLUP_GAP-24 | 0.343 | 9.8908E-05 | 0.08 | 3.76 |  |
| *QSug.uga-2B.2* | S2B_52675079 | SUG | BLUP_GAP-24 | 0.343 | 9.8908E-05 | 0.08 | 3.76 |  |
|  | S2B_52675082 | SUG | BLUP_GAP-24 | 0.343 | 9.89078E-05 | 0.08 | 3.76 |  |
| *QDm.uga-2B.2* | S2B_53295820 | DM | BLUP_GAP-25 | 0.480 | 5.80531E-05 | 0.21 | 3.39 |  |
|  | S2B_53295873 | DM | BLUP_GAP-25 | 0.477 | 6.89194E-05 | 0.21 | 3.33 |  |
| *QTdn.uga-2B* | S2B_224649603 | TDN | BLUP_GAP-25 | 0.209 | 5.5758E-05 | -0.83 | 5.12 |  |
| *QDm.uga-2B.3* | S2B_236592784 | DM | BLUP_All | 0.209 | 3.0691E-06 | -0.00 | 1.80 |  |
|  | S2B_236592794 | DM | BLUP_All | 0.209 | 3.0691E-06 | -0.00 | 1.80 |  |
|  | S2B_236592837 | DM | BLUP_All | 0.209 | 3.0691E-06 | -0.00 | 1.80 |  |
| *QDm.uga-2B.4* | S2B_245318621 | DM | BLUP_All | 0.218 | 5.1404E-05 | -0.00 | 4.49 |  |
|  | S2B_245318622 | DM | BLUP_All | 0.218 | 5.1404E-05 | -0.00 | 4.49 |  |
|  | S2B_245318638 | DM | BLUP_All | 0.218 | 5.1404E-05 | -0.00 | 4.49 |  |
|  | S2B_245318643 | DM | BLUP_All | 0.218 | 5.1404E-05 | -0.00 | 4.49 |  |
| *QDm.uga-2B.5* | S2B_339106848 | DM | BLUP_All | 0.224 | 2.8814E-05 | -0.00 | 2.50 |  |
|  | S2B_339106909 | DM | BLUP_All | 0.224 | 2.8814E-05 | -0.00 | 2.50 |  |
| *QDm.uga-2B.6* | S2B_374262947 | DM | BLUP_All | 0.224 | 4.9118E-05 | -0.00 | 2.23 |  |
|  | S2B_374262955 | DM | BLUP_All | 0.224 | 4.9118E-05 | -0.00 | 2.23 |  |
| *QDm.uga-2B.7* | S2B_419321038 | DM | BLUP_All | 0.212 | 7.7551E-05 | -0.00 | 4.64 |  |
|  | S2B_419321045 | DM | BLUP_All | 0.212 | 7.7551E-05 | -0.00 | 4.83 |  |
|  | S2B_419321055 | DM | BLUP_All | 0.212 | 7.7551E-05 | -0.00 | 4.61 |  |
| *QAdf.uga-2B* | S2B_797144696 | ADF | BLUP_All | 0.040 | 5.8012E-05 | -0.63 | 5.20 |  |
| *QNdf.uga-2B.1* | S2B_639015160 | NDF | BLUP_All | 0.302 | 3.4338E-05 | -0.27 | 8.74 |  |
| *QNdf.uga-2B.2* | S2B_640613229 | NDF | BLUP_All | 0.497 | 5.0315E-05 | 0.25 | 0.80 |  |
| *QDm.uga-2B.8* | S2B_548651156 | DM | BLUP_All | 0.212 | 4.3408E-05 | -0.00 | 3.21 |  |
|  | S2B_548651157 | DM | BLUP_All | 0.212 | 4.3408E-05 | -0.00 | 3.21 |  |
|  | S2B_548651179 | DM | BLUP_All | 0.212 | 4.3408E-05 | -0.00 | 3.21 |  |
|  | S2B_548651194 | DM | BLUP_All | 0.212 | 4.3408E-05 | -0.00 | 3.21 |  |
| *QSug.uga-2B.3* | S2B_617632042 | SUG | BLUP_GAP-25 | 0.026 | 6.9658E-05 | 0.63 | 7.26 |  |
|  | S2B_617632045 | SUG | BLUP_GAP-25 | 0.026 | 6.9658E-05 | 0.63 | 7.26 |  |
|  | S2B_617632046 | SUG | BLUP_GAP-25 | 0.026 | 6.9658E-05 | 0.63 | 7.26 |  |
|  | S2B_617632052 | SUG | BLUP_GAP-25 | 0.026 | 6.9658E-05 | 0.63 | 7.26 |  |
| *QNdf/Adf.uga-2B* | S2B_680431542 | NDF | BLUP_IHF-25 | 0.241 | 2.868E-05 | -1.22 | 2.43 |  |
|  | S2B_680431568 | NDF | BLUP_IHF-25 | 0.241 | 2.868E-05 | -1.22 | 2.19 |  |
|  | S2B_680431542 | ADF | BLUP_IHF-25 | 0.241 | 5.687E-05 | -1.10 | 2.68 |  |
|  | S2B_680431568 | ADF | BLUP_IHF-25 | 0.241 | 5.687E-05 | -1.10 | 2.19 |  |
| *QCp.uga-2B* | S2B_260239865 | CP | BLUP_IHF-25 | 0.072 | 7.58598E-05 | -0.36 | **9.76** |  |
| *QDm.uga-2B.9* | S2B_734339241 | DM | BLUP_All | 0.427 | 5.3413E-05 | 0.00 | 6.67 |  |
|  | S2B_734339252 | DM | BLUP_All | 0.427 | 5.3413E-05 | 0.00 | 6.67 |  |
|  | S2B_734339284 | DM | BLUP_All | 0.427 | 5.3413E-05 | 0.00 | 7.52 |  |
|  | S2B_734339292 | DM | BLUP_All | 0.427 | 5.3413E-05 | 0.00 | 6.67 |  |
| *QDm.uga-2B.10* | S2B_183200294 | DM | BLUP_All | 0.212 | 3.6683E-05 | -0.00 | 3.47 |  |
| *QDm.uga-2B.11* | S2B_562207106 | DM | BLUP_All | 0.212 | 1.5442E-05 | -0.00 | 4.08 |  |
| *QDm.uga-2B.12* | S2B_438459145 | DM | BLUP_All | 0.206 | 2.1831E-05 | -0.00 | 2.64 |  |
| *QDm.uga-2B.13* | S2B_291619677 | DM | BLUP_All | 0.209 | 2.2367E-05 | -0.00 | 3.26 |  |
| *QDm.uga-2B.14* | S2B_406888434 | DM | BLUP_All | 0.209 | 2.2367E-05 | -0.00 | 3.17 |  |
| *QCp.uga-2D* | S2D_9921979 | CP | BLUP_IHF-25 | 0.128 | 2.0867E-06 | -0.31 | **14.94** |  |
|  | S2D_9922024 | CP | BLUP_IHF-25 | 0.128 | 2.0867E-06 | -0.31 | **14.94** |  |
|  | S2D_9928132 | CP | BLUP_IHF-25 | 0.104 | 3.95753E-06 | -0.36 | **15.10** |  |
| *QCp.uga-2D.1* | S2D_20939495 | CP | BLUP_All | 0.319 | 0.0000168 | 0.09 | 5.04 |  |
|  | S2D_20939497 | CP | BLUP_All | 0.323 | 0.0000178 | 0.09 | 5.06 |  |
|  | S2D_20939525 | CP | BLUP_All | 0.319 | 0.0000168 | 0.09 | 5.04 |  |
|  | S2D_20939532 | CP | BLUP_All | 0.319 | 0.0000168 | 0.09 | 5.04 |  |
|  | S2D_21002547 | CP | BLUP_All | 0.314 | 0.0000211 | 0.09 | 4.00 |  |
| *QCp.uga-2D.2* | S2D_22347907 | CP | BLUP_All | 0.334 | 0.00000276 | 0.10 | 5.96 |  |
|  | S2D_22347911 | CP | BLUP_All | 0.334 | 0.00000276 | 0.10 | 5.96 |  |
|  | S2D_22347933 | CP | BLUP_All | 0.334 | 0.00000276 | 0.10 | 5.96 |  |
|  | S2D_22163614 | CP | BLUP_All | 0.331 | 0.00000539 | 0.09 | 6.84 |  |
|  | S2D_22264324 | CP | BLUP_All | 0.334 | 0.00000591 | 0.09 | 6.27 |  |
|  | S2D_22264330 | CP | BLUP_All | 0.334 | 0.00000591 | 0.09 | 6.27 |  |
|  | S2D_22264342 | CP | BLUP_All | 0.334 | 0.00000591 | 0.09 | 6.27 |  |
|  | S2D_22444692 | CP | BLUP_All | 0.331 | 0.00000572 | 0.09 | 6.68 |  |
|  | S2D_21556325 | CP | BLUP_All | 0.323 | 0.00000817 | 0.09 | 6.46 |  |
|  | S2D_21556338 | CP | BLUP_All | 0.323 | 0.00000817 | 0.09 | 6.46 |  |
|  | S2D_21556341 | CP | BLUP_All | 0.323 | 0.00000817 | 0.09 | 6.46 |  |
|  | S2D_21825683 | CP | BLUP_All | 0.326 | 0.00000667 | 0.10 | 6.08 |  |
|  | S2D_21825700 | CP | BLUP_All | 0.326 | 0.00000667 | 0.10 | 6.08 |  |
|  | S2D_22275927 | CP | BLUP_All | 0.328 | 0.0000103 | 0.09 | 5.60 |  |
|  | S2D_22275938 | CP | BLUP_All | 0.328 | 0.0000103 | 0.09 | 5.60 |  |
|  | S2D_22275945 | CP | BLUP_All | 0.328 | 0.0000103 | 0.09 | 5.60 |  |
|  | S2D_22275962 | CP | BLUP_All | 0.328 | 0.0000103 | 0.09 | 5.60 |  |
|  | S2D_22321001 | CP | BLUP_All | 0.326 | 0.0000112 | 0.09 | 4.96 |  |
|  | S2D_22321002 | CP | BLUP_All | 0.326 | 0.0000112 | 0.09 | 4.96 |  |
|  | S2D_22321026 | CP | BLUP_All | 0.326 | 0.0000112 | 0.09 | 4.96 |  |
|  | S2D_22321035 | CP | BLUP_All | 0.326 | 0.0000112 | 0.09 | 4.96 |  |
|  | S2D_22326701 | CP | BLUP_All | 0.328 | 0.00000841 | 0.09 | 5.73 |  |
|  | S2D_22335819 | CP | BLUP_All | 0.326 | 0.0000112 | 0.09 | 5.55 |  |
|  | S2D_22335840 | CP | BLUP_All | 0.326 | 0.0000112 | 0.09 | 4.96 |  |
|  | S2D_22352745 | CP | BLUP_All | 0.326 | 0.0000112 | 0.09 | 5.55 |  |
|  | S2D_22275583 | CP | BLUP_All | 0.334 | 0.0000117 | 0.09 | 5.74 |  |
|  | S2D_22275634 | CP | BLUP_All | 0.334 | 0.0000117 | 0.09 | 5.74 |  |
|  | S2D_22275646 | CP | BLUP_All | 0.334 | 0.0000117 | 0.09 | 5.74 |  |
|  | S2D_22110951 | CP | BLUP_All | 0.323 | 0.0000119 | 0.09 | 6.13 |  |
|  | S2D_22439610 | CP | BLUP_All | 0.328 | 0.0000124 | 0.09 | 5.69 |  |
|  | S2D_21785249 | CP | BLUP_All | 0.326 | 0.0000166 | 0.09 | 5.06 |  |
|  | S2D_21789928 | CP | BLUP_All | 0.323 | 0.0000154 | 0.09 | 5.08 |  |
|  | S2D_21789941 | CP | BLUP_All | 0.323 | 0.0000154 | 0.09 | 5.08 |  |
|  | S2D_21789942 | CP | BLUP_All | 0.323 | 0.0000154 | 0.09 | 5.08 |  |
|  | S2D_21782970 | CP | BLUP_All | 0.323 | 0.0000179 | 0.09 | 5.19 |  |
|  | S2D_21783003 | CP | BLUP_All | 0.323 | 0.0000179 | 0.09 | 5.19 |  |
|  | S2D_21510808 | CP | BLUP_All | 0.317 | 0.0000232 | 0.09 | 5.05 |  |
|  | S2D_22205102 | CP | BLUP_All | 0.294 | 0.0000244 | 0.10 | 5.77 |  |
|  | S2D_22205111 | CP | BLUP_All | 0.294 | 0.0000244 | 0.10 | 5.77 |  |
|  | S2D_21790002 | CP | BLUP_All | 0.323 | 0.0000255 | 0.09 | 4.76 |  |
|  | S2D_21790056 | CP | BLUP_All | 0.323 | 0.0000255 | 0.09 | 4.76 |  |
|  | S2D_22160686 | CP | BLUP_All | 0.334 | 0.0000278 | 0.09 | 8.22 |  |
|  | S2D_22171878 | CP | BLUP_All | 0.270 | 0.0000279 | 0.10 | 6.16 |  |
|  | S2D_22171881 | CP | BLUP_All | 0.270 | 0.0000279 | 0.10 | 6.16 |  |
|  | S2D_21535436 | CP | BLUP_All | 0.326 | 0.0000448 | 0.09 | 5.27 |  |
|  | S2D_21535446 | CP | BLUP_All | 0.326 | 0.0000448 | 0.09 | 5.82 |  |
|  | S2D_21535463 | CP | BLUP_All | 0.326 | 0.0000448 | 0.09 | 5.82 |  |
| *QAdf.uga-3A.1* | S3A_480442137 | ADF | BLUP_GAP-24 | 0.192 | 3.369E-05 | -0.22 | 8.52 |  |
| *QRfq.uga-3A* | S3A_60001784 | RFQ | BLUP_GAP-24 | 0.093 | 7.8424E-05 | 3.35 | 8.94 |  |
| *QSug.uga-3A* | S3A_724514751 | SUG | BLUP_GAP-25 | 0.105 | 9.42062E-05 | 0.32 | 5.53 |  |
| *QDm.uga-3A* | S3A_724527135 | DM | BLUP_GAP-25 | 0.038 | 1.89042E-05 | 0.76 | **11.00** |  |
| *QDm.uga-3A.1* | S3A_724514751 | DM | BLUP_GAP-25 | 0.105 | 7.02982E-05 | 0.31 | 5.90 |  |
| *QCp.uga-3A* | S3A_577990641 | CP | BLUP_IHF-25 | 0.128 | 3.46008E-05 | -0.26 | **15.42** |  |
| *QAdf.uga-3A.2* | S3A_534332254 | ADF | BLUP_IHF-25 | 0.483 | 9.5276E-05 | -0.36 | 3.66 |  |
| *QSug.uga-3B.1* | S3B_513097025 | SUG | BLUP_GAP-25 | 0.134 | 9.77852E-05 | 0.43 | 4.10 |  |
|  | S3B_513097038 | SUG | BLUP_GAP-25 | 0.134 | 9.77852E-05 | 0.43 | 4.10 |  |
|  | S3B_513097042 | SUG | BLUP_GAP-25 | 0.134 | 9.77852E-05 | 0.43 | 4.10 |  |
| *QRfq.uga-3B.1* | S3B_566641307 | RFQ | BLUP_GAP-25 | 0.134 | 3.80738E-05 | 0.43 | **10.72** |  |
|  |  | RFQ | BLUP_All | 0.041 | 5.50381E-05 | 0.08 | 7.65 |  |
|  | S3B_566480372 | RFQ | BLUP_All | 0.038 | 7.32474E-05 | 0.08 | 7.76 |  |
|  | S3B_566480398 | RFQ | BLUP_All | 0.038 | 7.32474E-05 | 0.08 | 7.76 |  |
| *QSug.uga-3B.2* | S3B_561366011 | SUG | BLUP_GAP-25 | 0.035 | 9.06223E-05 | 0.67 | 8.69 |  |
|  |  |  | BLUP_All | 0.035 | 9.575E-05 | 0.29 | 7.97 |  |
| *QRfq.uga-3B.2* | S3B_570524495 | RFQ | BLUP_GAP-25 | 0.041 | 3.80738E-05 | 1.77 | **10.72** |  |
|  |  |  | BLUP_All | 0.041 | 5.50381E-05 | 0.08 | 7.65 |  |
| *QSug.uga-3B.3* | S3B_181072778 | SUG | BLUP_GAP-25 | 0.017 | 3.1735E-05 | -0.95 | 9.42 |  |
| *QNdf/Adf.uga-3B* | S3B_666328969 | NDF | BLUP_IHF-25 | 0.041 | 9.5171E-06 | -1.48 | 3.26 |  |
|  |  | ADF | BLUP_All | 0.041 | 2.5138E-05 | -0.63 | 3.99 |  |
|  |  |  | BLUP_IHF-25 | 0.041 | 4.1571E-05 | -1.25 | 3.75 |  |
|  |  |  | BLUP_GAP-25 | 0.041 | 8.4434E-05 | -1.09 | 3.62 |  |
| *QRfq.uga-3B.3* | S3B_556649844 | RFQ | BLUP_All | 0.035 | 2.65621E-05 | 0.09 | 9.24 |  |
| *QRfq.uga-3B.4* | S3B_559885495 | RFQ | BLUP_All | 0.035 | 2.86856E-05 | 0.09 | 9.01 |  |
| *QDm.uga-3D* | S3D_555274375 | DM | BLUP_GAP-24 | 0.003 | 1.1407E-12 | -0.81 | **17.29** |  |
| *QCp.uga-4A.1* | S4A_712451436 | CP | BLUP_IHF-25 | 0.049 | 7.34916E-05 | -0.39 | **12.67** |  |
|  | S4A_711229076 | CP | BLUP_IHF-25 | 0.046 | 9.40973E-05 | -0.39 | **11.92** |  |
|  | S4A_711229113 | CP | BLUP_IHF-25 | 0.046 | 9.40973E-05 | -0.39 | **12.28** |  |
|  | S4A_711331984 | CP | BLUP_IHF-25 | 0.046 | 9.40973E-05 | -0.39 | **12.28** |  |
|  | S4A_711417921 | CP | BLUP_IHF-25 | 0.046 | 9.40973E-05 | -0.39 | **9.64** |  |
|  | S4A_711664614 | CP | BLUP_IHF-25 | 0.046 | 9.40973E-05 | -0.39 | **12.28** |  |
|  | S4A_711904190 | CP | BLUP_IHF-25 | 0.046 | 9.40973E-05 | -0.39 | **12.28** |  |
|  | S4A_711971234 | CP | BLUP_IHF-25 | 0.046 | 9.40973E-05 | -0.39 | **12.28** |  |
|  | S4A_711971270 | CP | BLUP_IHF-25 | 0.046 | 9.40973E-05 | -0.39 | **12.28** |  |
| *QRfq.uga-4A* | S4A_725532798 | RFQ | BLUP_GAP-25 | 0.224 | 3.84041E-05 | 0.99 | **11.23** |  |
|  | S4A_725532813 | RFQ | BLUP_GAP-25 | 0.224 | 3.84041E-05 | 0.99 | **11.23** |  |
| *QDm.uga-4A.1* | S4A_699898062 | DM | BLUP_GAP-24 | 0.279 | 2.2433E-08 | -0.06 | 4.64 |  |
| *QDm.uga-4A.2* | S4A_640698671 | DM | BLUP_GAP-24 | 0.465 | 8.6468E-05 | -0.03 | 2.80 |  |
| *QCp.uga-4A.2* | S4A_405677816 | CP | BLUP_GAP-24 | 0.073 | 9.7237E-06 | -0.58 | **11.35** |  |
| *QTL.small.46* | S4A_707136165 | CP | BLUP_GAP-25 | 0.125 | 9.0101E-05 | 0.34 | 7.73 |  |
| *QCp.uga-4A.3* | S4A_12623190 | CP | BLUP_IHF-25 | 0.026 | 8.12723E-05 | -0.52 | **13.67** |  |
| *QRfq.uga-4B.1* | S4B_6076831 | RFQ | BLUP_All | 0.5 | 9.25333E-05 | 0.04 | **9.79** |  |
|  | S4B_6076862 | RFQ | BLUP_All | 0.5 | 9.25333E-05 | 0.04 | 8.57 |  |
|  | S4B_6076872 | RFQ | BLUP_All | 0.5 | 9.25333E-05 | -0.04 | 8.57 |  |
|  | S4B_6076892 | RFQ | BLUP_All | 0.5 | 9.25333E-05 | 0.04 | 8.57 |  |
| *QSug.uga-4B* | S4B_670762786 | SUG | BLUP_GAP-25 | 0.326 | 2.55577E-05 | 0.22 | **9.54** |  |
|  | S4B_670800146 | SUG | BLUP_GAP-25 | 0.323 | 5.68869E-05 | 0.22 | **11.04** |  |
| *QRfq.uga-4B.2* | S4B_2845950 | RFQ | BLUP_All | 0.5 | 9.40252E-05 | 0.04 | **9.79** |  |
| *QNdf.uga-4D* | S4D_4606119 | NDF | BLUP_GAP-25 | 0.105 | 1.2279E-05 | -0.89 | 7.75 |  |
| *QRfq.uga-5A* | S5A_30060588 | RFQ | BLUP_GAP-24 | 0.047 | 8.2422E-05 | 4.13 | 6.17 |  |
| *QAdl.uga-5A* | S5A_682890015 | ADL | BLUP_GAP-25 | 0.142 | 3.5098E-05 | 0.04 | **11.22** |  |
| *QNdf.uga-5A* | S5A_4676039 | NDF | BLUP_GAP-25 | 0.346 | 9.6903E-05 | 0.59 | 6.64 |  |
| *QTdn.uga-5A* | S5A_489631352 | TDN | BLUP_IHF-25 | 0.366 | 5.1533E-05 | -0.29 | 6.32 |  |
| *QSug.uga-5A* | S5A_48882371 | SUG | BLUP_IHF-25 | 0.087 | 8.8471E-05 | 0.37 | 8.39 |  |
| *QAdl/Tdn.uga-5A* | S5A_19997583 | ADL | BLUP_All | 0.026 | 8.9551E-05 | 0.07 | 0.52 |  |
|  |  | TDN |  | 0.026 | 8.9551E-05 | 0.07 | 8.28 |  |
| *QAdl.uga-5B* | S5B_588509484 | ADL | BLUP_IHF-25 | 0.195 | 9.5106E-05 | 0.02 | 8.52 |  |
|  | S5B_588509535 | ADL | BLUP_IHF-25 | 0.189 | 6.2496E-05 | 0.02 | 8.14 |  |
| *QAdl.uga-5B.1* | S5B_605517698 | ADL | BLUP_IHF-25 | 0.113 | 4.7201E-05 | 0.02 | **11.54** |  |
|  | S5B_605518747 | ADL | BLUP_IHF-25 | 0.113 | 4.8472E-05 | 0.02 | **10.53** |  |
| *QAdf.uga-5B.1* | S5B_626709654 | ADF | BLUP_GAP-25 | 0.177 | 5.2452E-05 | 0.50 | 5.69 |  |
|  | S5B_626709683 | ADF | BLUP_GAP-25 | 0.177 | 5.2452E-05 | 0.50 | 5.69 |  |
| *QDm.uga-5B* | S5B_596931041 | DM | BLUP_GAP-24 | 0.108 | 5.3857E-05 | -0.06 | 3.86 |  |
| *QAdf.uga-5B.2* | S5B_626183011 | ADF | BLUP_GAP-25 | 0.201 | 9.5743E-05 | 0.45 | 6.08 |  |
| *QAdf.uga-5B.3* | S5B_628039272 | ADF | BLUP_GAP-25 | 0.189 | 8.4953E-05 | 0.46 | 6.63 |  |
| *QAdl.uga-5B.2* | S5B_486483108 | ADL | BLUP_GAP-25 | 0.483 | 8.7536E-05 | 0.03 | **10.53** |  |
| *QAdl.uga-5B.3* | S5B_607020013 | ADL | BLUP_IHF-25 | 0.110 | 4.4172E-05 | 0.02 | **11.32** |  |
| *QTdn.uga-5D* | S5D_392075408 | TDN | BLUP_GAP-24 | 0.151 | 7.4296E-05 | 0.27 | 2.29 |  |
| *QAdl.uga-6B* | S6B_6242037 | ADL | BLUP_GAP-25 | 0.041 | 2.9139E-05 | 0.08 | **12.97** |  |
|  | S6B_6242055 | ADL | BLUP_GAP-25 | 0.041 | 2.9139E-05 | 0.08 | **12.97** |  |
| *QAdl.uga-6B.1* | S6B_139231751 | ADL | BLUP_GAP-25 | 0.081 | 8.4581E-05 | 0.07 | 4.07 |  |
|  | S6B_139231757 | ADL | BLUP_GAP-25 | 0.081 | 8.4581E-05 | 0.07 | 4.07 |  |
| *QSug/Tdn.uga-6B* | S6B_168773568 | SUG | BLUP_GAP-25 | 0.311 | 9.50129E-05 | -0.22 | 8.47 |  |
|  | S6B_168773601 | SUG | BLUP_GAP-25 | 0.334 | 2.29482E-05 | -0.24 | 9.18 |  |
|  |  | TDN | BLUP_GAP-25 | 0.334 | 5.1348E-05 | -0.21 | 8.02 |  |
| *QRfq.uga-6B.1* | S6B_483865496 | RFQ | BLUP_GAP-25 | 0.334 | 5.07881E-05 | -0.97 | 5.08 |  |
|  | S6B_483865536 | RFQ | BLUP_GAP-25 | 0.334 | 5.07881E-05 | -0.97 | 5.93 |  |
|  | S6B_483865538 | RFQ | BLUP_GAP-25 | 0.334 | 5.07881E-05 | -0.97 | 5.93 |  |
|  | S6B_483865545 | RFQ | BLUP_GAP-25 | 0.334 | 5.07881E-05 | -0.97 | 5.93 |  |
| *QRfq.uga-6B.2* | S6B_486225291 | RFQ | BLUP_GAP-25 | 0.328 | 8.94766E-05 | -0.95 | 8.30 |  |
|  | S6B_486225321 | RFQ | BLUP_GAP-25 | 0.328 | 8.94766E-05 | -0.95 | 6.11 |  |
| *QRfq.uga-6B.3* | S6B_486764902 | RFQ | BLUP_GAP-25 | 0.326 | 8.98144E-05 | -0.95 | 8.14 |  |
|  | S6B_486764935 | RFQ | BLUP_GAP-25 | 0.326 | 8.98144E-05 | -0.95 | 5.52 |  |
|  | S6B_487031016 | RFQ | BLUP_GAP-25 | 0.326 | 9.31571E-05 | -0.94 | 8.11 |  |
|  | S6B_487031019 | RFQ | BLUP_GAP-25 | 0.326 | 9.31571E-05 | -0.94 | 5.60 |  |
| *QRfq.uga-6B.4* | S6B_493117261 | RFQ | BLUP_GAP-25 | 0.340 | 1.94692E-05 | -1.01 | 5.66 |  |
|  | S6B_492193353 | RFQ | BLUP_GAP-25 | 0.331 | 3.43141E-05 | -0.99 | 8.62 |  |
|  | S6B_492857027 | RFQ | BLUP_GAP-25 | 0.323 | 3.55436E-05 | -0.98 | 5.47 |  |
|  | S6B_492508735 | RFQ | BLUP_GAP-25 | 0.331 | 5.54764E-05 | -0.96 | 9.00 |  |
| *QRfq.uga-6B.5* | S6B_516559638 | RFQ | BLUP_GAP-25 | 0.334 | 3.22107E-05 | -0.98 | 5.57 |  |
|  | S6B_516693627 | RFQ | BLUP_GAP-25 | 0.326 | 9.51473E-05 | -0.95 | 5.99 |  |
| *QRfq.uga-6B.6* | S6B_517693881 | RFQ | BLUP_GAP-25 | 0.326 | 8.86648E-05 | -0.95 | 5.03 |  |
|  | S6B_517693930 | RFQ | BLUP_GAP-25 | 0.326 | 8.86648E-05 | -0.95 | 5.03 |  |
| *QRfq.uga-6B.7* | S6B_519412209 | RFQ | BLUP_GAP-25 | 0.326 | 6.07077E-05 | -0.96 | 6.57 |  |
|  | S6B_519412226 | RFQ | BLUP_GAP-25 | 0.326 | 6.07077E-05 | -0.96 | 5.62 |  |
|  | S6B_519412251 | RFQ | BLUP_GAP-25 | 0.326 | 6.07077E-05 | -0.96 | 5.62 |  |
| *QRfq.uga-6B.8* | S6B_527759913 | RFQ | BLUP_GAP-25 | 0.328 | 5.6673E-05 | -0.97 | 5.82 |  |
|  | S6B_527759952 | RFQ | BLUP_GAP-25 | 0.328 | 5.6673E-05 | -0.97 | 5.82 |  |
|  | S6B_527825477 | RFQ | BLUP_GAP-25 | 0.323 | 6.54994E-05 | -0.97 | 5.02 |  |
|  | S6B_527825494 | RFQ | BLUP_GAP-25 | 0.326 | 8.60729E-05 | -0.95 | 5.27 |  |
| *QRfq.uga-6B.9* | S6B_532043734 | RFQ | BLUP_GAP-25 | 0.328 | 5.35456E-05 | -0.98 | 5.03 |  |
|  | S6B_532043730 | RFQ | BLUP_GAP-25 | 0.331 | 6.6721E-05 | -0.96 | 5.83 |  |
| *QCp.uga-6B.1* | S6B_691137110 | CP | BLUP_GAP-24 | 0.078 | 2.78871E-05 | 0.49 | 8.89 |  |
| *QRfq.uga-6B.10* | S6B_484538781 | RFQ | BLUP_GAP-25 | 0.331 | 5.38795E-05 | -0.97 | 6.33 |  |
| *QRfq.uga-6B.11* | S6B_485310414 | RFQ | BLUP_GAP-25 | 0.319 | 5.5171E-05 | -0.98 | 8.31 |  |
| *QRfq.uga-6B.12* | S6B_481855711 | RFQ | BLUP_GAP-25 | 0.323 | 7.69567E-05 | -0.96 | 4.99 |  |
| *QRfq.uga-6B.13* | S6B_528915436 | RFQ | BLUP_GAP-25 | 0.332 | 8.11661E-05 | -0.96 | 5.70 |  |
| *QRfq.uga-6B.14* | S6B_529738129 | RFQ | BLUP_GAP-25 | 0.319 | 8.68794E-05 | -0.95 | 5.68 |  |
| *QCp.uga-6B.2* | S6B_4852064 | CP | BLUP_IHF-25 | 0.116 | 3.30295E-05 | -0.34 | 0.63 |  |
| *QDm.uga-6D.1* | S6D_68707263 | DM | BLUP_IHF-25 | 0.047 | 6.9127E-05 | -0.12 | 4.26 |  |
|  | S6D_68707270 | DM | BLUP_IHF-25 | 0.047 | 6.9127E-05 | -0.12 | 6.86 |  |
| *QDm.uga-6D.2* | S6D_69219136 | DM | BLUP_IHF-25 | 0.047 | 6.9127E-05 | -0.12 | 4.26 |  |
|  | S6D_69219206 | DM | BLUP_IHF-25 | 0.047 | 6.9127E-05 | -0.12 | 4.26 |  |
|  | S6D_69307498 | DM | BLUP_IHF-25 | 0.047 | 6.9127E-05 | -0.12 | 4.26 |  |
| *QDm.uga-6D.3* | S6D_79588697 | DM | BLUP_IHF-25 | 0.049 | 5.7252E-05 | -0.12 | 4.43 |  |
|  | S6D_79614070 | DM | BLUP_IHF-25 | 0.047 | 6.9127E-05 | -0.12 | 4.26 |  |
| *QDm.uga-6D.4* | S6D_81114877 | DM | BLUP_IHF-25 | 0.047 | 6.9127E-05 | -0.12 | 4.26 |  |
|  | S6D_81114880 | DM | BLUP_IHF-25 | 0.047 | 6.9127E-05 | -0.12 | 4.26 |  |
|  | S6D_81114883 | DM | BLUP_IHF-25 | 0.047 | 6.9127E-05 | -0.12 | 4.26 |  |
|  | S6D_81114889 | DM | BLUP_IHF-25 | 0.047 | 6.9127E-05 | -0.12 | 4.26 |  |
| *QDm.uga-6D.5* | S6D_81659451 | DM | BLUP_IHF-25 | 0.047 | 6.9127E-05 | -0.12 | 4.26 |  |
|  | S6D_81749865 | DM | BLUP_IHF-25 | 0.047 | 6.9127E-05 | -0.12 | 6.86 |  |
|  | S6D_81920593 | DM | BLUP_IHF-25 | 0.047 | 6.9127E-05 | -0.12 | 4.26 |  |
| *QDm.uga-6D.6* | S6D_84308691 | DM | BLUP_IHF-25 | 0.047 | 6.9127E-05 | -0.12 | 4.26 |  |
|  | S6D_84308701 | DM | BLUP_IHF-25 | 0.047 | 6.9127E-05 | -0.12 | 4.26 |  |
| *QDm.uga-6D.7* | S6D_85041305 | DM | BLUP_IHF-25 | 0.047 | 6.9127E-05 | -0.12 | 6.86 |  |
|  | S6D_85041325 | DM | BLUP_IHF-25 | 0.047 | 6.9127E-05 | -0.12 | 6.86 |  |
|  | S6D_85108083 | DM | BLUP_IHF-25 | 0.047 | 6.9127E-05 | -0.12 | 6.86 |  |
| *QDm.uga-6D.8* | S6D_77864626 | DM | BLUP_IHF-25 | 0.049 | 6.7306E-05 | -0.12 | 7.07 |  |
| *QDm.uga-6D.9* | S6D_75283108 | DM | BLUP_IHF-25 | 0.047 | 6.9127E-05 | -0.12 | 4.26 |  |
| *QDm.uga-6D.10* | S6D_75873588 | DM | BLUP_IHF-25 | 0.047 | 6.9127E-05 | -0.12 | 6.86 |  |
| *QDm.uga-6D.11* | S6D_76615971 | DM | BLUP_IHF-25 | 0.047 | 6.9127E-05 | -0.12 | 4.26 |  |
| *QDm.uga-6D.12* | S6D_85906363 | DM | BLUP_IHF-25 | 0.047 | 6.9127E-05 | -0.12 | 4.26 |  |
| *QDm.uga-6D.13* | S6D_86544010 | DM | BLUP_IHF-25 | 0.047 | 6.9127E-05 | -0.12 | 4.26 |  |
| *QCp.uga-6D* | S6D_109883550 | CP | BLUP_All | 0.282 | 0.00000447 | 0.11 | 6.41 |  |
| *QDm/Sug.uga-7A* | S7A_3765553 | DM | BLUP_GAP-24 | 0.093 | 2.0572E-14 | -0.11 | 6.40 |  |
|  | S7A_2974209 | SUG | BLUP_GAP-25 | 0.157 | 3.91912E-05 | 0.27 | **10.64** |  |
|  | S7A_3571986 | SUG | BLUP_GAP-25 | 0.186 | 5.00578E-05 | 0.26 | 6.49 |  |
|  | S7A_3426596 | SUG | BLUP_GAP-25 | 0.189 | 5.67863E-05 | 0.27 | **10.57** |  |
|  | S7A_3426616 | SUG | BLUP_GAP-25 | 0.189 | 5.67863E-05 | 0.27 | **10.57** |  |
|  | S7A_3426620 | SUG | BLUP_GAP-25 | 0.189 | 5.67863E-05 | 0.27 | **10.57** |  |
|  | S7A_3426628 | SUG | BLUP_GAP-25 | 0.189 | 5.67863E-05 | 0.27 | **10.57** | |
| *QTdn/Adl.uga-7B* | S7B_59821499 | TDN | BLUP_All | 0.235 | 8.4112E-05 | -0.03 | 6.89 | |
|  |  | ADL | BLUP_All | 0.235 | 8.4112E-05 | -0.03 | 4.49 | |
| *QDm.uga-7D* | S7D_531373369 | DM | BLUP_GAP-24 | 0.108 | 1.1443E-05 | 0.06 | 4.51 | |

**Supplementary Figure 1:** Scatter plot showing linkage disequilibrium (LD) across the wheat genome and each chromosome. The r^2^ of pair of single nucleotide polymorphisms (SNP) was plotted against the physical distance in base pairs (bp). The numbers in the blue show the LD decay distance at the LD population threshold of 0.2.

**Distance (bd)**

**LD (r^2^)**

**Supplementary Figure 2:** Comparison of four different GWAS models for acid detergent fiber (ADF) (top) and dry matter (DM) (bottom) from combined environment data of all three individual environments. GLM, generalized linear model; MLM, mixed linear model; MLMM, multiple loci mixed model; FarmCPU, fixed and random model circulating probability unification;
